# Deletion of *F4L* (ribonucleotide reductase) in vaccinia virus produces a selective oncolytic virus and promotes anti‐tumor immunity with superior safety in bladder cancer models

**DOI:** 10.15252/emmm.201607296

**Published:** 2017-03-13

**Authors:** Kyle G Potts, Chad R Irwin, Nicole A Favis, Desmond B Pink, Krista M Vincent, John D Lewis, Ronald B Moore, Mary M Hitt, David H Evans

**Affiliations:** ^1^ Department of OncologyFaculty of Medicine and DentistryUniversity of AlbertaEdmontonABCanada; ^2^ Li Ka Shing Institute of VirologyFaculty of Medicine and DentistryUniversity of AlbertaEdmontonABCanada; ^3^ Cancer Research Institute of Northern Alberta (CRINA)Faculty of Medicine and DentistryUniversity of AlbertaEdmontonABCanada; ^4^ Department of Medical Microbiology & ImmunologyFaculty of Medicine and DentistryUniversity of AlbertaEdmontonABCanada; ^5^ Department of Anatomy & Cell BiologyFaculty of Medicine and DentistryUniversity of Western OntarioLondonONCanada; ^6^ Department of SurgeryFaculty of Medicine and DentistryUniversity of AlbertaEdmontonABCanada

**Keywords:** bladder cancer, immunotherapy, oncolytic virus, ribonucleotide reductase, vaccinia virus, Cancer, Genetics, Gene Therapy & Genetic Disease, Urogenital System

## Abstract

Bladder cancer has a recurrence rate of up to 80% and many patients require multiple treatments that often fail, eventually leading to disease progression. In particular, standard of care for high‐grade disease, Bacillus Calmette–Guérin (BCG), fails in 30% of patients. We have generated a novel oncolytic vaccinia virus (VACV) by mutating the *F4L* gene that encodes the virus homolog of the cell‐cycle‐regulated small subunit of ribonucleotide reductase (RRM2). The *F4L*‐deleted VACVs are highly attenuated in normal tissues, and since cancer cells commonly express elevated RRM2 levels, have tumor‐selective replication and cell killing. These *F4L*‐deleted VACVs replicated selectively in immune‐competent rat AY‐27 and xenografted human RT112‐luc orthotopic bladder cancer models, causing significant tumor regression or complete ablation with no toxicity. It was also observed that rats cured of AY‐27 tumors by VACV treatment developed anti‐tumor immunity as evidenced by tumor rejection upon challenge and by *ex vivo* cytotoxic T‐lymphocyte assays. Finally, *F4L*‐deleted VACVs replicated in primary human bladder cancer explants. Our findings demonstrate the enhanced safety and selectivity of *F4L*‐deleted VACVs, with application as a promising therapy for patients with BCG‐refractory cancers and immune dysregulation.

## Introduction

Over 90% of cases of bladder cancer are subtyped as urothelial cell carcinomas. When first diagnosed, about 80% of these cases are classified as non‐muscle‐invasive bladder cancer (NMIBC) [reviewed in (Anastasiadis & de Reijke, 2012; Potts *et al*, 2012; Delwar *et al*, 2016)], but unfortunately up to 80% of these patients will experience a recurrence within 5 years of initial treatment (van Rhijn *et al*, 2009). Current standards of care for low‐risk patients include surgery and intravesical chemotherapy (Shen *et al*, 2008). The high‐grade (Ta, T1, or carcinoma *in situ*) tumors are most likely to recur, and treatment for these patients includes surgery often followed by intravesical therapy with Bacillus Calmette–Guérin (BCG) (Shen *et al*, 2008). The side effects of BCG treatment include a risk of infection, cystitis, and prostatitis, and it can be hazardous for immunocompromised patients (Lamm *et al*, 1992). Moreover, about 30% of patients fail BCG therapy leaving cystectomy as the next common treatment option (Zlotta *et al*, 2009). How BCG works is poorly understood and it may simply be a pro‐inflammatory agent (Redelman‐Sidi *et al*, 2014). There is no evidence to suggest that BCG generates protective anti‐tumor immunity and this may partly explain the high rate of treatment failure (Biot *et al*, 2012). In fact, there has been very little improvement in the treatment of high‐grade NMIBC in the last 10–20 years and recurrence after BCG therapy is still one of the most significant problems in the management of bladder cancer (Downs *et al*, 2015). This highlights the urgent need for safer and more reliable bladder‐sparing approaches.

Oncolytic viruses are intended to replicate selectively in, and kill, cancer cells while sparing normal tissues [reviewed in (Potts *et al*, 2012; Russell *et al*, 2012; Kaufman *et al*, 2015)]. Some of the many cell pathways that affect virus replication are those that regulate cell proliferation and DNA replication, processes that are critically dependent upon deoxynucleoside triphosphate (dNTP) production (Aye *et al*, 2015). The rate‐limiting step in dNTP biosynthesis is the *de novo* reduction of ribonucleoside diphosphates (rNDPs) to deoxyribonucleoside diphosphates (dNDPs) by the enzyme ribonucleoside diphosphate reductase (RNR) (Nordlund & Reichard, 2006). Since DNA virus replication requires dNTPs, this requirement for RNR activity provides an important biological feature that can be used to target DNA viruses to cancer cells.

Many viruses exhibit oncolytic properties and a modified herpesvirus, Talimogene laherparepvec (T‐Vec), recently received US clinical approval (Andtbacka *et al*, 2015). Vaccinia virus (VACV) has also been studied extensively as an oncolytic agent, with JX‐594 (Pexa‐Vec) having completed multiple phase II trials (Hwang *et al*, 2011; Heo *et al*, 2013; Cripe *et al*, 2015; Park *et al*, 2015). VACV is a large double‐stranded DNA virus that efficiently infects many different cell types and encodes many of the proteins required for robust replication in normal cells (McFadden, 2005). These proteins include thymidine kinase (TK; the *J2R* gene product) and both large (RRM1; *I4L* gene product) and small (RRM2; *F4L* gene product) subunits of the heterodimeric RNR complex (Slabaugh *et al*, 1988). The virus‐encoded components of RNR complex with each other and can form chimeras with cellular homologs (Hendricks & Mathews, 1998; Gammon *et al*, 2010). Most oncolytic VACVs reported to date encode mutations in *J2R* and little research has been conducted to determine whether mutating the RNR genes (Fend *et al*, 2016) might also produce advantageous oncolytic properties. The *F4L* gene is an important determinant of VACV virulence and viruses lacking *F4L* (∆*F4L*) are attenuated *in vivo* whereas ∆*I4L* mutants are not (Gammon *et al*, 2010). The fact that cellular RRM2 is cell‐cycle‐regulated whereas RRM1 is constitutively expressed can perhaps explain this observation (Eriksson *et al*, 1984) and leads to the prediction that a ∆*F4L* virus should replicate selectively in dividing cancer cells. This complementation‐based strategy might be especially useful for treating more aggressive bladder cancers since increased levels of cellular RRM2 predict a poorer prognosis (Morikawa *et al*, 2010).

Here we describe pre‐clinical studies showing that VACV can be used safely as an intravesical treatment for NMIBC. We find that *F4L*‐deleted VACVs retain much of their cytotoxicity and replication proficiency in bladder cancer cells. *F4L*‐deleted VACVs also safely and effectively clear bladder tumors in animal models and induce a durable anti‐tumor immunity. These findings highlight the potential for using a *F4L*‐deleted VACV in treating bladder cancer, especially in patients who have failed BCG treatment or are immunosuppressed.

## Results

### Growth of VACV Δ*F4L* and Δ*J2R* mutants *in vitro*

**Figure EV1:**
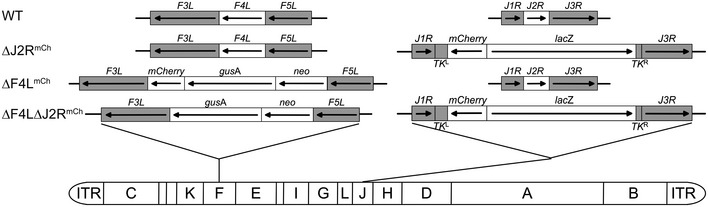
Genomic map of vaccinia virus constructs Viruses were generated from the VACV Western Reserve strain. Viral thymidine kinase is encoded by the *J2R* gene. Subunit 2 of viral ribonucleotide reductase is encoded by the *F4L* gene. *neo*, neomycin gene; *gusA*, β‐glucuronidase gene; *lacZ*, β‐galactosidase gene; ITR, inverted terminal repeat; *TK* ^L^, viral thymidine kinase gene left homology; *TK* ^R^, viral thymidine kinase gene right homology; and WT, wild‐type.

**Figure 1:**
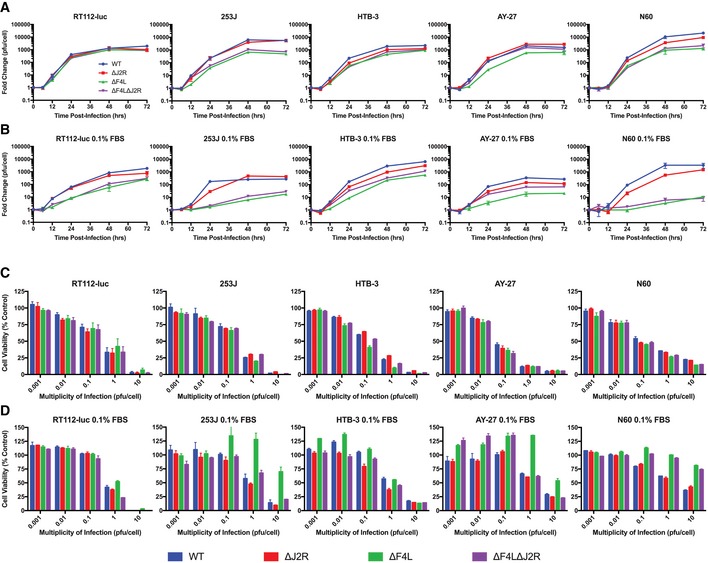
∆*F4L*∆*J2R* VACV retains much of the replication proficiency and cytotoxicity of WT VACV in bladder cancer cells A, BGrowth curves for the indicated VACV strains in subconfluent human bladder cancer cell lines, a rat bladder cancer cell line (AY‐27), and a normal human skin fibroblast line (N60). The cells were infected with 0.03 PFU/cell. (A) Panel of cells grown under normal serum conditions. (B) Panel of cells grown under low (0.1%) serum conditions. Cultures were harvested at the indicated times and titered on BSC‐40 cells.C, DSurvival of cell lines infected *in vitro* with the indicated VACV strains. Subconfluent cells were infected at the indicated multiplicities of infection (in PFU/cell). Uninfected cells were used as control. (C) Panel of cells grown under normal serum conditions. (D) Panel of cells grown under low (0.1%) serum conditions. The cells were incubated with resazurin to assess viability 3 days post‐infection relative to uninfected control cells.Data information: Mean ± SEM is shown. For (A) and (B), data represent at least two independent lysates titered in duplicate. For (C) and (D) *n *≥* *3.

**Figure EV2:**
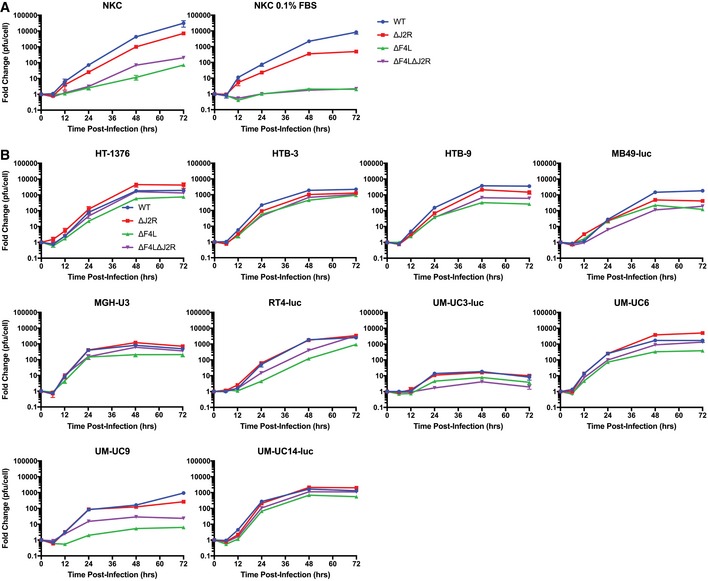
∆*F4L*∆*J2R* VACV retains much of the replication proficiency of wild‐type VACV in bladder cancer cells Growth curves for the indicated VACV mutants or WT VACV. Subconfluent cells were infected at a multiplicity of infection of 0.03 PFU/cell. Cultures were harvested at the indicated times and titered on BSC‐40 cells. 
Normal human kidney epithelial cells grown under normal serum conditions (left) and 0.1% FBS (right).Panel of human bladder cancer cell lines (exception: MB49‐luc, murine urothelial carcinoma) cultured *in vitro* with 10% FBS.Data information: Mean ± SEM is shown and data represent at least two independent lysates titered in duplicate.

Homologous recombination was used to disrupt the VACV (strain Western Reserve) *F4L* and *J2R* loci as shown in Fig EV1. Thirteen out of fifteen bladder cancer cell lines grown under high serum conditions (10%) supported robust virus replication, exceptions being UM‐UC3‐luc and UM‐UC9 cells (Figs 1A and EV2). Under low serum conditions (0.1%), the wild‐type (WT) and ∆*J2R* VACV grew as well as was seen in 10% serum. Although viruses lacking *F4L* also replicated efficiently in most of the cancer cell lines under low serum conditions, the ∆*F4L* VACVs grew poorly in 253J and AY‐27 cells (Fig 1B). Most importantly, compared to WT, growth of ∆*F4L*∆*J2R* and ∆*F4L* VACVs in low serum was reduced > 4,000‐fold in the NKC (normal epithelial kidney) line and > 250‐fold in N60 (normal fibroblast) cell line, whereas the growth of ∆*J2R* VACV was only marginally reduced compared to WT in the NKC and N60 cells under the same low serum conditions (Figs 1 and EV2).

**Figure EV3:**
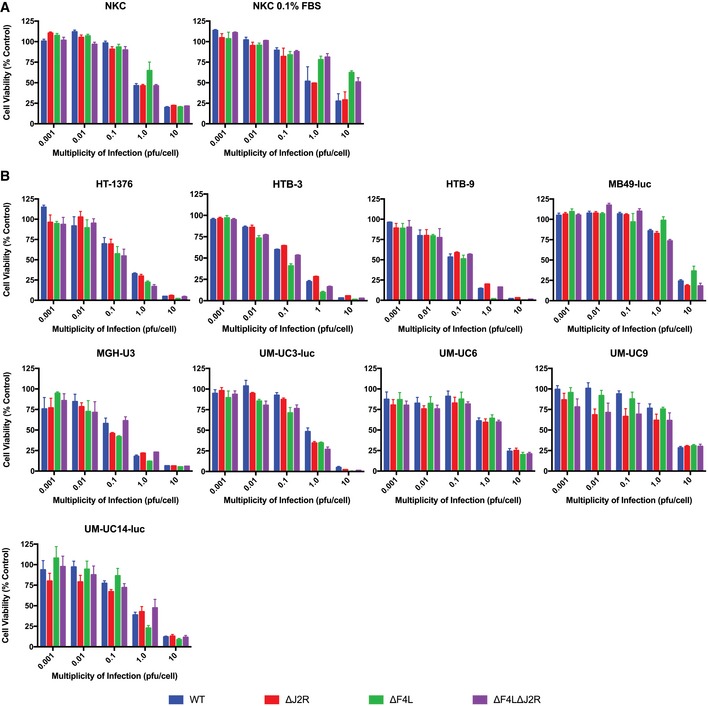
∆*F4L*∆*J2R* VACV retains much of the cytotoxicity of wild‐type VACV in bladder cancer cells Survival of cell lines infected *in vitro* with the indicated VACV strains. Subconfluent cells were infected at the indicated multiplicities of infection (in PFU/cell). Uninfected cells were used as control.
Normal human kidney epithelial cells grown under normal serum conditions (left) and 0.1% FBS (right).Panel of human bladder cancer cell lines (exception: MB49‐luc, murine urothelial carcinoma) cultured *in vitro* with 10% FBS. The cells were incubated with resazurin to assess viability 3 days post‐infection relative to uninfected control cells. Uninfected cells were used as control.Data information: Mean ± SEM is shown and *n *≥* *3.

**Figure EV4:**
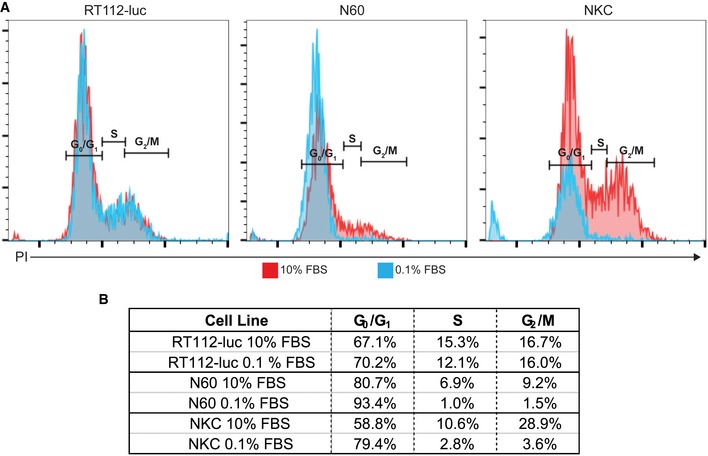
Non‐tumorigenic cells have reduced S‐phase population when grown under lower serum conditions Cell cycle analyses of indicated cell lines.
Indicated cells lines were grown in media supplemented with either 10% FBS or 0.1% FBS for 48 h, and then cell cycle distribution was monitored by flow cytometry after PI staining. Red traces indicate cells grown in 10% FBS and blue traces indicate cells grown in 0.1% FBS.Analysis of cell cycle phase distribution.

The effect of VACV on cell survival was determined using a resazurin‐based viability assay. Similar to growth of virus under high serum conditions (Fig 1A), no dramatic difference in the efficiency of virus‐mediated cell killing was seen among the different viruses under high serum conditions (Figs 1C and EV3). However, under low serum conditions, both N60 normal skin fibroblasts and NKC epithelial kidney cells became relatively resistant to ∆*F4L* and ∆*F4L*∆*J2R* VACV killing. Interestingly, in low serum conditions, 253J and AY‐27 cancer cells were still highly susceptible to killing by ∆*F4L*∆*J2R* VACV (Fig 1D), even though virus replication was attenuated. This was a specific property of the ∆*F4L*∆*J2R* virus; 253J and AY‐27 cells were still relatively resistant to the ∆*F4L* VACV. Finally, both N60 and NKC cells grown in 0.1% fetal bovine serum (FBS) showed a low proportion of cells in S‐phase whereas the proportion of RT112‐luc cells in S‐phase remained high (Fig EV4), suggesting that proliferation status under our low serum growth conditions may mimic the proliferation status of normal and tumor tissues *in vivo*. These data indicate that the mutant VACVs, in particular ∆*F4L*∆*J2R* VACV, retained much of the cytotoxic capabilities and replication proficiency of WT virus in bladder cancer cells but do not replicate in non‐dividing cells.

### Nucleotide biosynthetic proteins are elevated in bladder cancer cells

**Figure 2:**
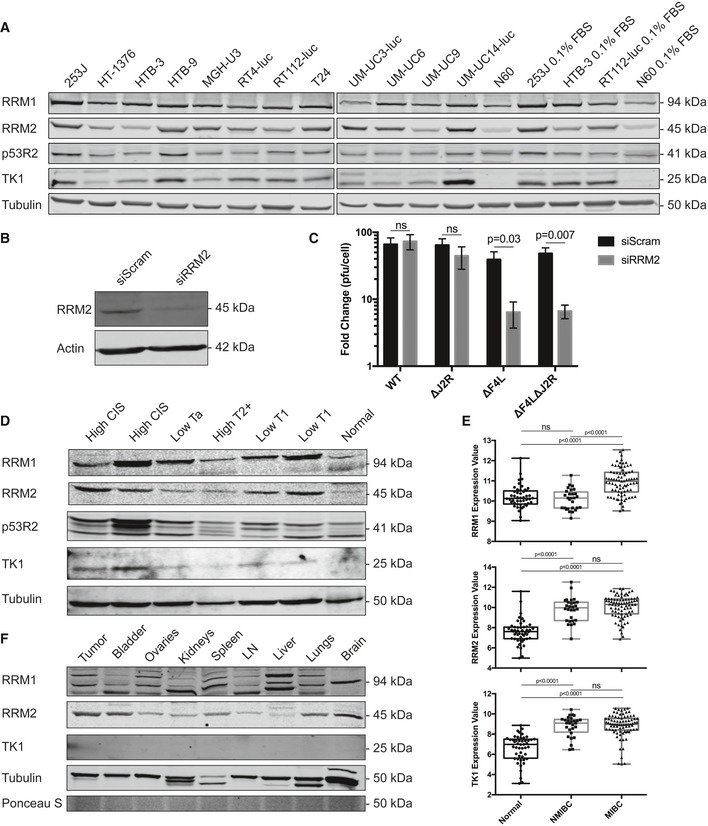
Elevated levels of proteins catalyzing nucleotide biosynthesis in bladder cancer cell lines and primary human tumor lysates Western blot showing RRM1, RRM2, p53R2, and TK1 expression in human bladder cancer cell lines and N60 normal human fibroblasts. β‐tubulin is shown as a loading control.siRNA depletion of RRM2 in HeLa cells 3 days post‐transfection as determined by Western blot analysis.Growth of the indicated VACV strains in subconfluent HeLa cells. The cells were treated for 24 h with a scrambled control siRNA (“Scram”) or an *RRM2*‐targeted siRNA and then infected with the indicated viruses at 0.03 PFU/cell. The cultures were harvested 2 days later and titered on BSC‐40 cells.Western blot showing RRM1, RRM2, p53R2, and TK1 expression levels in human primary tumor tissues and adjacent normal urothelium. β‐tubulin is shown as a loading control.Analysis of *RRM1*,* RRM2*, and *TK1* expression levels from publicly available patient bladder cancer microarray data (NMIBC: non‐muscle‐invasive bladder cancer; MIBC: muscle‐invasive bladder cancer). Data points denote log_2_‐transformed MAS5.0 normalized values. The box limits represent the upper and lower quartiles. The median is marked by the horizontal line inside the box. The whiskers extend to the highest and lowest observed values.Western blot showing RRM1, RRM2, and TK1 expression in rat AY‐27 bladder tumor tissue and the indicated normal tissues. β‐tubulin and Ponceau S staining are shown as loading controls. In all Western blots, equal amounts of total protein (30 μg) were assayed.Data information: Mean ± SEM is shown. For (C) *n* = 4 and significance was determined by multiple *t‐*test. Microarray data in (E) were analyzed using RStudio (v0.98.501) and significance analysis was performed using a one‐way ANOVA followed by Tukey's HSD. Western blots are representative of at least two or three independent experiments.

One might expect that ∆*F4L* and/or ∆*J2R* strains would depend upon complementation from cellular RRM2 and TK1, respectively, to provide the dNTPs required for virus replication. Some limited data suggested that ∆*F4L* VACVs do grow better in cells expressing higher levels of RRM2 (Gammon *et al*, 2010). To examine this matter in more detail, the levels of proteins catalyzing nucleotide biosynthesis were quantified in a panel of human bladder cancer cell lines and in normal N60 fibroblasts under 10 and 0.1% serum conditions (Fig 2A and Appendix Fig S1). Western blots showed a general elevation in the levels of cellular RRM1, RRM2, and TK1 in cancer cell lines compared to normal cells (Appendix Fig S1). The abundance of the DNA damage‐inducible form of R2, p53R2, did not significantly differ between the cancer cell lines and in normal skin fibroblasts.

To demonstrate *F4L*‐deleted VACVs' dependence on cellular RRM2, we tested whether knockdown of RRM2 in HeLa cells would prevent VACV replication. Efficient knockdown was achieved following transfection with *RRM2*‐specific siRNAs, as confirmed by Western blot analysis (Fig 2B). The cells were then infected with the different VACVs and virus yield measured by plaque assay (Fig 2C). There was a significant reduction in ∆*F4L* and ∆*F4L*∆*J2R* VACV replication in cells with RRM2 knockdown, while WT or ∆*J2R* VACV replication was unaffected.

We also examined expression of nucleotide metabolism proteins in samples isolated from primary human bladder tumors and from normal bladder urothelium. Western blot analysis showed elevated expression of both RRM1 and RRM2 in the tumor tissues relative to the normal urothelium (Fig 2D). Additionally, TK1 was only detectable in the tumor lysates, with two of these showing high expression levels, and only minimal expression in the remaining lysates. As in cultured cells, p53R2 expression was not specifically associated with tumors. These observations were generally corroborated by gene expression data obtained from primary tumor samples previously analyzed by Sanchez‐Carbayo *et al* (2006). Reanalysis of these data showed that *RRM2* and *TK1* expression were significantly increased in both NMIBC and muscle‐invasive bladder cancers (MIBC) when compared to the normal urothelium (Fig 2E). In contrast, *RRM1* was only significantly over‐expressed in MIBC.

The expression level of these same proteins was also measured in different tissues recovered from an orthotopic rat AY‐27 bladder cancer model (Fig 2F). We detected high RRM2 expression in tumor tissue as well as normal bladder. RRM1 did not appear elevated in tumors compared to normal tissues, and very little TK1 expression was detected in any of the tissues.

### VACVs encoding *F4L* and *J2R* mutations safely clear human bladder tumor xenografts

**Figure 3:**
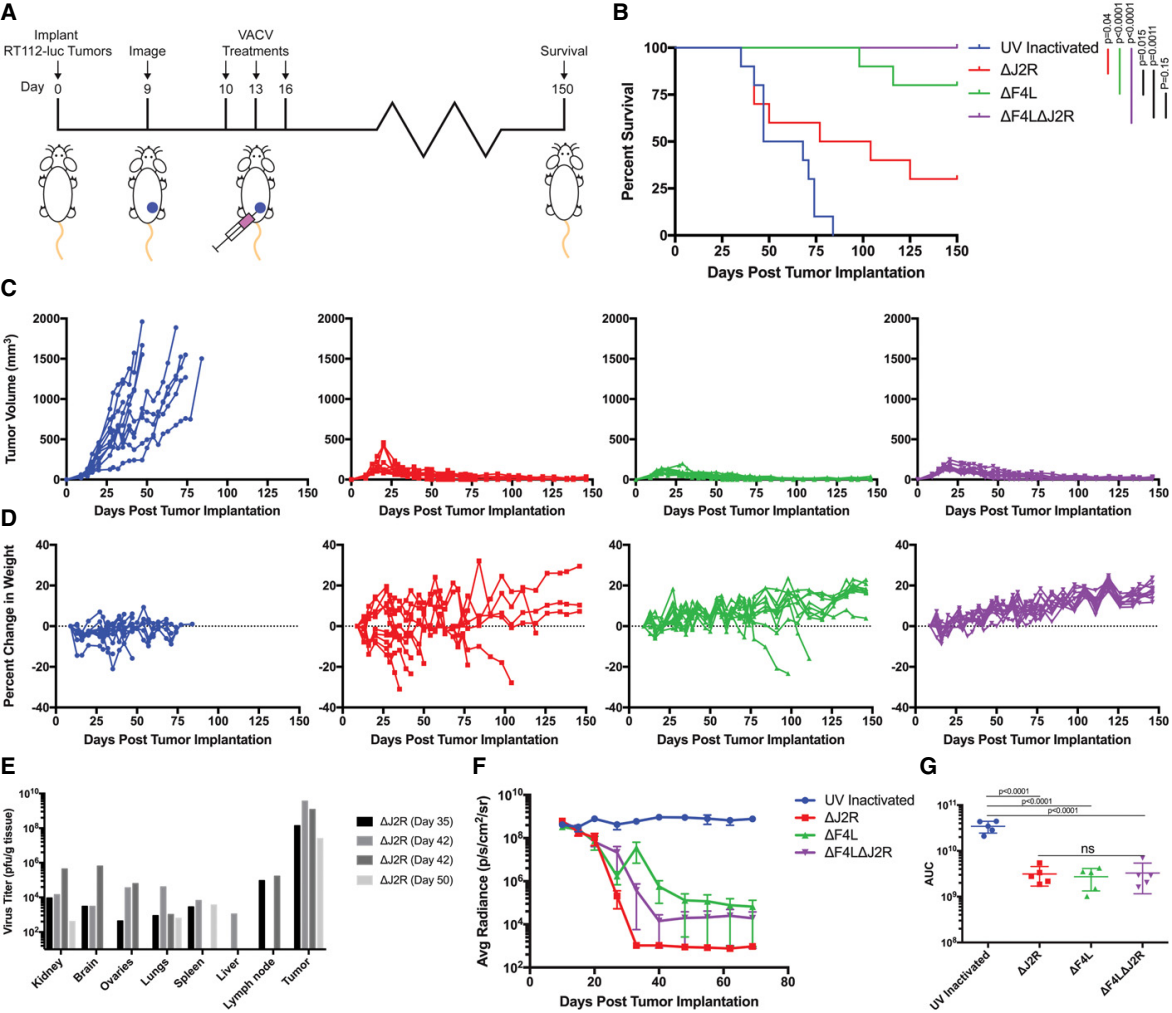
∆*F4L*∆*J2R* VACV safely and effectively clears subcutaneous human RT112‐luc xenografted tumors Experimental scheme. Balb/c nude mice were injected with 2 × 10^6^ RT112‐luc cells in the left flank at day zero. Then, 10^6^ PFU of UV‐inactivated, ∆*J2R*, ∆*F4L*, or ∆*F4L*∆*J2R* VACV were injected into the tumors on days 10, 13, and 16 post‐implantation.Overall survival of immunocompromised mice bearing RT112‐luc flank tumors following treatment with the indicated viruses (*n* = 10 per group).Growth of individual virus‐treated RT112‐luc tumors. Legend as in (B).Analysis of individual animal body weights plotted as mean change in body weight relative to day 10. Legend as in (B).VACV titers in tissues taken from animals euthanized due to toxicity (note: only mice that had detectable (4/10) virus as determined by plaque assay are shown).Quantification of average luminescence (an indication of live tumor cells) from bladder tumors corresponding to (B).Area under the curve (AUC) calculation from the data in (F) (*n *=* *5 per group).Data information: Mean ± SEM is shown. Animal survival was analyzed by log‐rank (Mantel–Cox) test in (B). One‐way ANOVA followed by Tukey's multiple comparison test was used in (G). For luciferase quantification in (F) and AUG calculations in (G) *n *=* *5 representative animals.

The safety and oncolytic activity of the mutant VACVs was tested in xenograft models of human bladder cancer. These models were established by subcutaneous or orthotopic implantation of luciferase‐expressing human RT112 cells (RT112‐luc) in Balb/c immune‐deficient mice. In the first study, we injected three doses of virus, each comprising 10^6^ PFU of ∆*J2R*, ∆*F4L*, ∆*F4L*∆*J2R*, or UV‐inactivated VACV as a control, directly into subcutaneous RT112‐luc tumors (Fig 3A). An mCherry signal, indicative of virus replication, was detected in all mice before the third live virus injection (Appendix Fig S2) and all animals treated with live virus showed significantly prolonged survival compared to those treated with UV‐inactivated VACV (Fig 3B). Both ∆*F4L* and ∆*F4L*∆*J2R* VACV significantly increased survival compared to animals treated with the ∆*J2R* strain (*P* = 0.015 and *P* = 0.001, respectively). Tumor growth was controlled in all animals treated with live viruses as determined by caliper measurements (Fig 3C), and by luciferase detection (Fig 3F and G, and Appendix Fig S3).

The ∆*J2R* virus showed strong anti‐tumor activity, but this was only achieved with significant toxicity in Balb/c immune‐deficient mice. Seven of ten ∆*J2R* VACV‐treated mice were euthanized due to excessive weight loss (Fig 3D). The mice euthanized prior to day 50 exhibited significant viremia at endpoint (Fig 3E) whereas the mice euthanized at later time points had acquired systemic *Staphylococcus aureus* infections. These, as well as other ∆*J2R* VACV‐treated mice, exhibited transient and spontaneously resolving dermal pox lesions at sites distal to the tumor injection site, which may have provided a route for the bacterial infection. In contrast, all mice treated with ∆*F4L*∆*J2R* VACV were completely cured of their RT112‐luc tumors and continued to gain weight throughout the experiment (Fig 3D). We saw no signs of toxicity or virus lesions in this treatment group. A pilot experiment using a subcutaneous UM‐UC3‐luc xenograft model also suggested strong oncolytic activity from ∆*F4L*∆*J2R* VACV (Appendix Fig S4) even though UM‐UC3‐luc cells supported only limited VACV growth *in vitro* (Fig EV2).

**Figure 4:**
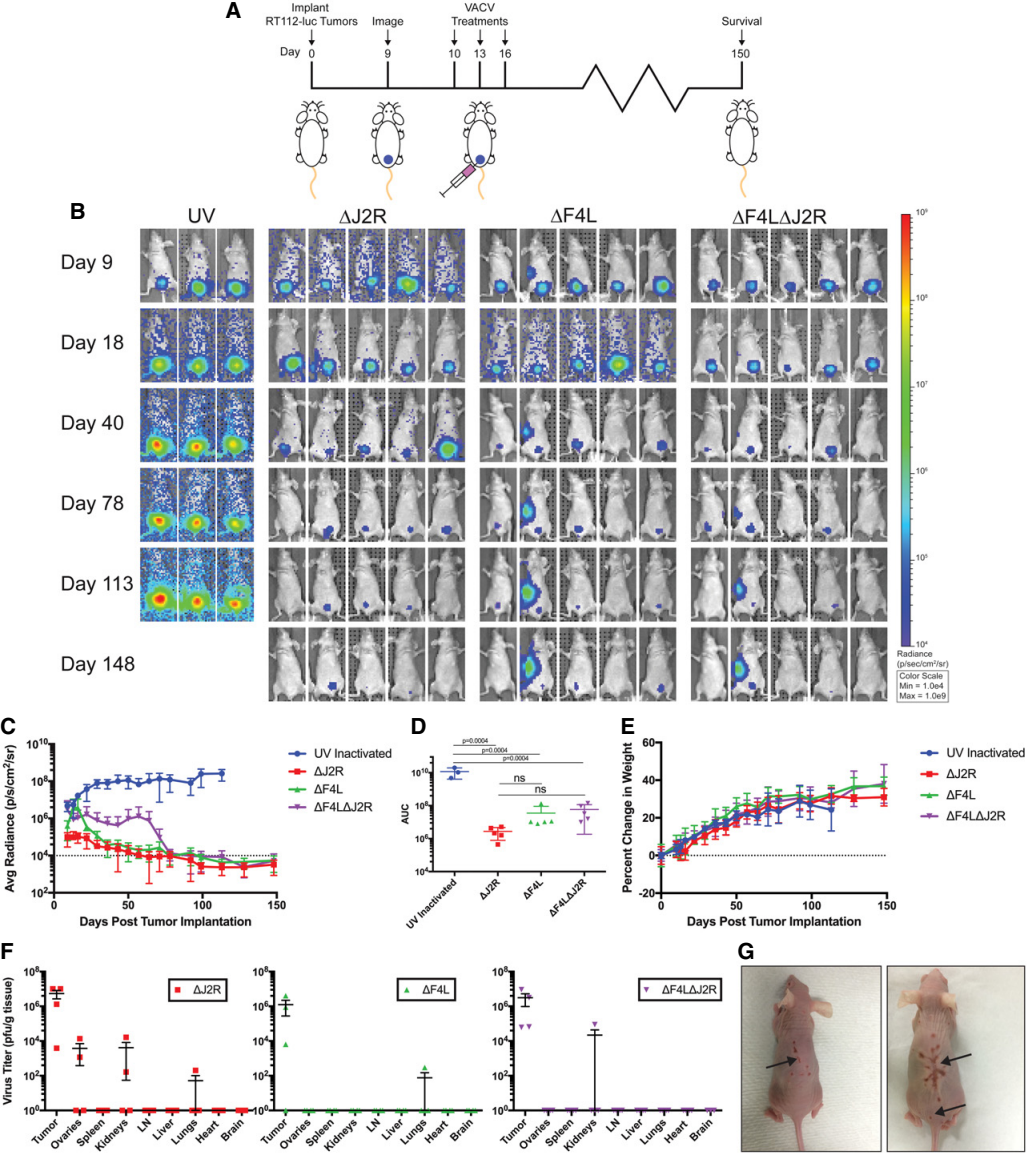
∆*F4L*∆*J2R* VACV safely and effectively clears orthotopic human RT112‐luc xenografted tumors Experimental scheme. Balb/c nude mice were instilled with 2 × 10^6^ RT112‐luc cells on day zero. Mice were imaged for luciferase following luciferin injection on day 9 to verify tumor implantation. On each of days 10, 13, and 16 post‐tumor implantations, 10^6^ PFU of UV‐inactivated, ∆*J2R*, ∆*F4L*, or ∆*F4L*∆*J2R* VACV was instilled into the bladder and left in‐dwell for 1 h. *N *=* *5 per group.Representative luminescence images from animals bearing orthotopic RT112‐luc tumors and treated with VACVs.Quantification of average luminescence, the dashed line indicates limit of detection.Area under the curve calculation from the data in (C).Analysis of individual animal body weights plotted as mean change in body weight.Virus titers in tissues on day 19. Organs were harvested, homogenized, and the virus was titered on BSC‐40 cells with *n *=* *4 mice per group.Representative images of lesions on two mice taken approximately 125 days post‐tumor implantation. Arrows indicate lesions.Data information: Mean ± SEM is shown. For (C), (D), and (E) *n *=* *3 for UV‐inactivated and 5 for live viruses. One‐way ANOVA followed by Tukey's multiple comparison test was used in (D).

We next developed a new orthotopic RT112‐luc xenograft model to replace the KU7 model that was recently shown to have been contaminated with HeLa cells (Jäger *et al*, 2013). The treatment scheme is shown in Fig 4A. Bioluminescence images show a continuous increase in luciferase signal from tumors treated with UV‐inactivated virus, and a decline in signal from all live‐VACV‐treated animals, with most tumors eventually being cleared (Fig 4B–D). To measure virus distribution after intravesical treatment, we euthanized mice 3 days after the last virus instillation and measured virus titers in tumors and other organs. We detected little or no spread of the ∆*F4L* or ∆*F4L*∆*J2R* virus to other organs (Fig 4F). In one animal, significant levels of ∆*F4L*∆*J2R* VACV were detected in the kidney, but this coincided with a luciferase signal indicating tumor spread to this site, demonstrating the tumor selectivity of this virus. In contrast, ∆*J2R* VACV was detected in several organs in treated mice (Fig 4F). Delivering the virus directly into the bladder caused no toxicity as judged by animal weights (Fig 4E). However, as was seen in the subcutaneous model, most of the ∆*J2R*‐treated mice developed pox lesions on their backs (Fig 4G).

Finally, we tested efficacy and toxicity when virus was administered by intravenous injection in a subcutaneous RT112‐luc xenograft tumor model. ∆*J2R* VACV replication was detectable at the tumor site by day 20 (Appendix Fig S5, but this virus caused significant toxicity and showed no significant survival benefit over UV‐inactivated virus by log‐rank test (Appendix Fig S6A–D). In contrast, there was a significant increase in the survival of mice treated with the ∆*F4L* (*P* = 0.0015) and ∆*F4L*∆*J2R* (*P* = 0.013) viruses (Appendix Fig S6A and B). The ∆*F4L*∆*J2R* group exhibited virus‐encoded mCherry fluorescence at the tumor site by day 27 (Appendix Fig S6), while this signal was not seen until day 40 in the ∆*F4L* group.

### VACV mutants clear syngeneic orthotopic rat bladder tumors

**Figure 5:**
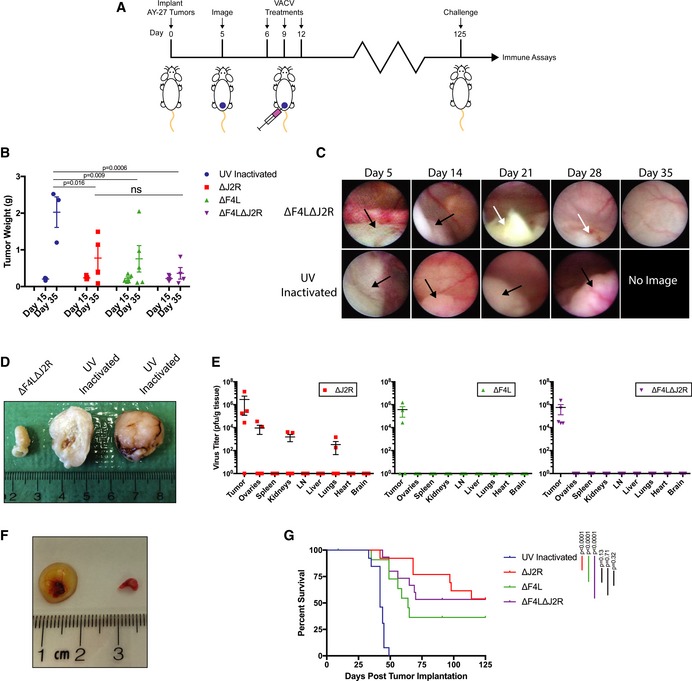
∆*F4L*∆*J2R* VACV safely clears rat orthotopic AY‐27 syngeneic tumors and generates protective anti‐tumor immunity Experimental scheme. Rats were instilled in the bladder with 3 × 10^6^ AY‐27 cells on day zero and cystoscoped on day 5 to verify tumor engraftment. Then, 3 × 10^8^ PFU of UV‐inactivated, ∆*J2R*, ∆*F4L*, or ∆*F4L*∆*J2R* VACV were instilled into the bladder of each rat on each of days 6, 9, and 12.Tumor weight from animals euthanized on days 15 and 35 (*n *=* *5 for each day).Representative cystoscope images of the bladders of a ∆*F4L*∆*J2R* virus‐treated rat and a UV‐inactivated virus‐treated rat on days 5, 14, 21, 28, and 35. Black arrow indicates tumor and white arrow indicates necrotic tumor tissues.Images of rat bladders treated with ∆*F4L*∆*J2R* VACV (left image) or UV‐inactivated VACV (center and right images) on days 6, 9, and 12, and then excised on day 35. The tumor treated with UV‐inactivated virus has been cut in half. The center sample shows the tumor interior; the right sample shows the exterior.Virus titers in tissues taken from animals euthanized on day 15 post‐implantations. Organs were harvested and homogenized and then lysates were titered on BSC‐40 cells. Data for each organ represent *n *=* *5 rats per group.Ovaries from rats euthanized 15 days post‐tumor implantations and 3 days following final treatment with ∆*J2R* VACV (left) or ∆*F4L*∆*J2R* VACV (right).Overall survival of immunocompetent rats bearing AY‐27 bladder tumors following treatment with the indicated VACVs. Data represent combined survival of two independent experiments (*n *=* *12–15).Data information: Mean ± SEM is shown. Two‐way ANOVA followed by Tukey's multiple comparison test was used in (B). Animal survival was analyzed by log‐rank (Mantel–Cox) test in (G).

**Figure 6:**
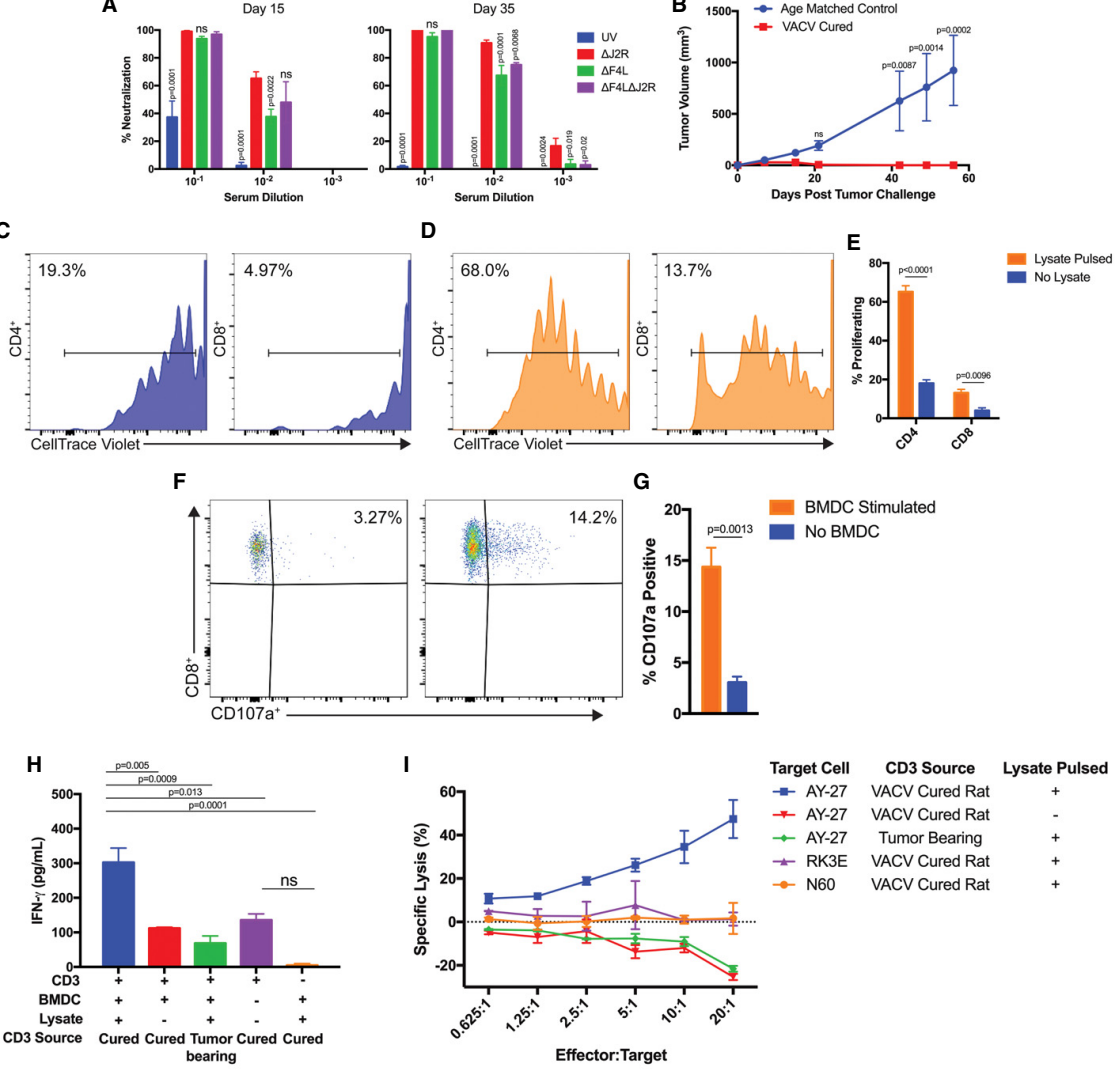
VACV activates immune responses in rats bearing AY‐27 bladder tumors AVACV‐neutralizing antibodies were measured in virus‐treated rats 15 and 35 days after implantation (*n *=* *4–5 rats per group).BProtection from subcutaneous tumor challenge after virus‐induced tumor clearance. AY‐27 cells were implanted in the flanks of cured rats (*n *=* *6) and naïve age‐matched control rats (*n *=* *4).C–ET‐cell proliferation after co‐culturing with bone marrow‐derived dendritic cells (BMDCs). CD4^+^ and CD8^+^ cells were co‐cultured with BMDCs and proliferation assayed with CellTrace Violet. The representative plots show CD4^+^ and CD8^+^ T‐cell proliferation after co‐culture with either mock‐pulsed (C) or with tumor‐lysate‐pulsed BMDCs (D). Panel (E) shows the percentage of CD4^+^ and CD8^+^ T cells that proliferated in response to BMDC stimulation (*n *=* *3).F, G
*Ex vivo* upregulation of CD107a by CD8^+^ T cells from challenged rats. (F) CD3^+^ cells were incubated +/− BMDCs for 1 h in the presence of anti‐CD107a antibody, incubated for 5 h with monensin and brefeldin A, and then stained with anti‐CD4 and anti‐CD8 antibodies. Events were gated for viable CD8^+^ T cells. Panel (G) shows the percentage of CD107a^+^ CD8^+^ T cells +/− BMDC stimulation (*n *=* *3).HIFN‐γ released after 24‐h co‐culture of CD3^+^ cells with BMDCs (*n *=* *3–5).IT cells activated *ex vivo* by tumor‐lysate‐pulsed DCs are cytotoxic. After 6 days of co‐culture with BMDC, CD3^+^ cells were incubated for 18 h with 10,000 target cells and at different effector‐to‐target ratios. Lysis was determined by LDH assay. RK3E are normal rat kidney cells (*n *=* *2–3 performed in duplicate).Data information: Mean ± SEM is shown. Two‐way ANOVA followed by Tukey's multiple comparison test was used in (A), (B), and (H). For (A), significance was determined against the ∆*J2R* group. Two‐tailed Student's *t‐*test was used in (E) and (G).

The mutant VACVs were also tested using an orthotopic immune‐competent AY‐27 rat bladder cancer model. AY‐27 tumors resemble high‐grade urothelial cell carcinoma in both morphology and tumor biology, providing an excellent model of human bladder cancer (Xiao *et al*, 1999). Animals were treated by intravesical instillation with 3 × 10^8^ PFU of live ∆*J2R*, ∆*F4L*, or ∆*F4L*∆*J2R* VACV, or UV‐inactivated virus (Fig 5A). By day 35, there was a significant reduction in the growth rate of all tumors treated with live virus (Fig 5B–D). Following ∆*F4L*∆*J2R* VACV treatment, representative cystoscopic images of the rat bladders revealed tumor necrosis and tumor elimination with little or no inflammation in normal urothelium (Fig 5C). On day 15 after tumor implantation (3 days after final virus instillation), ∆*F4L* and ∆*F4L*∆*J2R* VACVs could only be detected in the tumor, whereas ∆*J2R* VACV was found in the tumor and in the ovaries, kidneys, and lungs (Fig 5E). Of particular concern was the apparent development of cysts on the ovaries of some rats (Fig 5F). However, none of the animals treated with any virus showed signs of overt toxicity. The most important result of this study is that all live VACV treatments significantly increased survival (*P* < 0.001) when compared to the UV‐inactivated control (Fig 5G). It is noteworthy that although nine of the ∆*J2R* VACV‐treated animals appeared to be tumor‐free by day 75, three of these rats later developed rapidly growing recurrent tumors. This was not seen with either ∆*F4L* or ∆*F4L*∆*J2R* VACV as far out as 125 days into the study. Interestingly, we also observed significantly higher levels of anti‐VACV antibodies in animals treated with ∆*J2R* VACV relative to the *F4L*‐deleted VACVs (Fig 6A).

### Cured animals develop protective anti‐tumor immunity

To test whether animals with complete tumor responses had developed anti‐tumor immunity, we implanted fresh AY‐27 cells in the bladders of eleven surviving VACV‐treated animals and all cured animals rejected tumor implantation (Appendix Fig S7). Systemic anti‐tumor immunity was also tested by implanting fresh AY‐27 tumor cells subcutaneously in the flanks of six cured ∆*F4L*∆*J2R*‐treated animals. Again, all cured rats were protected from tumor development whereas significant tumor growth (*P* < 0.001) was seen in the age‐matched controls (Fig 6B).

The cellular anti‐tumor immune response was examined in cured animals that were resistant to tumor challenge. One hundred days after subcutaneous challenge with AY‐27 cells, cured ∆*F4L*∆*J2R*‐treated rats were euthanized, the spleens were removed, and CD3^+^ cells were isolated. The CD3^+^ cells were stimulated with bone marrow‐derived dendritic cells (BMDCs) previously pulsed with AY‐27 tumor lysate to enable antigen presentation (Bachleitner‐Hofmann *et al*, 2002). Both splenic CD3^+^CD4^+^ and CD3^+^CD8^+^ cells proliferated when stimulated by lysate‐pulsed BMDCs (Fig 6C–E). To confirm activation of the CD8^+^ cells, the CD3^+^CD8^+^ population was examined for expression of the CD107a marker (Betts *et al*, 2003). There were significantly more CD8^+^CD107a^+^ cells following stimulation with lysate‐pulsed BMDCs (Fig 6F and G). These stimulated CD3^+^ cells also exhibited elevated secretion of IFN‐γ (Fig 6H) and killed AY‐27 tumor cells, but not normal RK3E (F344 Fischer rat kidney) or N60 fibroblast cells (Fig 6I). Significantly, CD3^+^ cells from AY‐27‐tumor‐bearing rats that had never been exposed to VACV could not kill AY‐27 cells. Collectively, these data show that VACV treatment generated a durable tumor‐antigen‐specific cytotoxic T‐cell response in the AY‐27 rat model.

### VACV can replicate in primary tumor cell cultures and in tumor explants *ex vivo*

**Figure 7:**
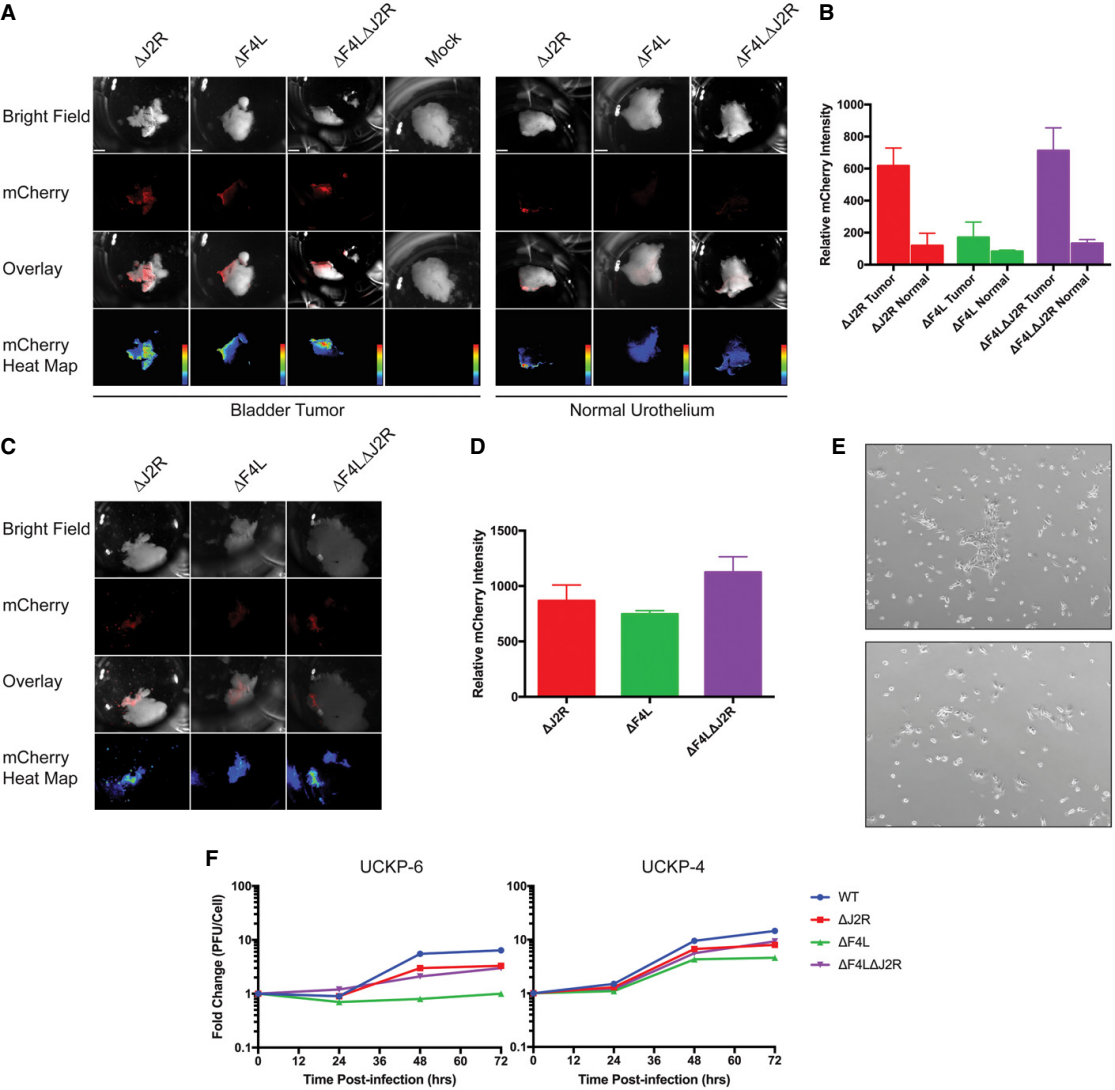
VACV infects and selectively replicates in primary bladder tumor tissue Viruses encoding mCherry fluorescent protein were used to infect primary low‐grade T1 (UCKP‐6) and normal urothelial tissue samples from patients undergoing transurethral resection of bladder tumors. Tissues were infected with 10^6^ PFU of the indicated viruses using buffered saline as a negative control (mock). The images from top to bottom represent a white light tissue image, mCherry signal, an overlay, and a heat map image showing mCherry expression, respectively (scale bars = 1 mm).Quantification of the mCherry expression in panel (A) at 24 h post‐infection. Mock‐infected cells were used as background correction. Represent one primary tissue that was analyzed as described in Data Information.
*Ex vivo* infection of high‐grade T2 (UCKP‐4) bladder tumor as in (A).Quantification of the mCherry expression in panel (C) at 24 h post‐infection. Represent one primary tissue that was analyzed as described in Data Information..Representative (4×) microscope pictures of the UCKP‐4 (top) and UCKP‐6 (bottom) primary cell cultures used in (F).Growth of the indicated VACV strains in UCKP‐4 primary human bladder cancer cultures. The cells were infected at 0.03 PFU/cell, harvested at the indicated times, and titered on BSC‐40 cells. Data represent single lysates titered in duplicate.Data information: Quantification in (B) and (D) shows the mean mCherry signal intensity (value per pixel) over the area of a given tumor sample. Data are shown as the mean ± SEM of these intensities over the tumor area.

We also examined whether ∆*F4L* and/or ∆*F4L*∆*J2R* VACV could replicate in human bladder cancers in either primary cell cultures or tumor explants. Explanted tissues from a low‐grade T1 tumor (UCKP‐6), a high‐grade T2^+^ tumor (UCKP‐4), and normal urothelium were infected with 10^6^ PFU of each VACV, and replication was detected using a virus‐encoded mCherry reporter (Fig 7A–D). In the low‐grade tumor, the ∆*F4L*∆*J2R* and ∆*J2R* VACVs produced nearly identical mCherry signals, while ∆*F4L* VACV produced a much lower signal (Fig 7A and B). In high‐grade tissues, all viruses produced nearly identical mCherry signals (Fig 7C and D). All viruses had minimal fluorescence in normal urothelium (Fig 7A). We used the same low‐ and high‐grade tumor samples to establish monolayer cell cultures (Fig 7E and F). The ∆*F4L*∆*J2R* VACV grew to the same level as ∆*J2R* VACV in both low‐ and high‐grade primary tumor cells, whereas the ∆*F4L* VACV grew more poorly in the low‐grade UCKP‐6 culture, just as was seen in the tissue explants. Collectively, these data support the hypothesis that the ∆*F4L*∆*J2R* VACV could be used to treat both high‐ and low‐grade bladder cancer.

### VACV can infect and replicate in BCG‐resistant bladder cancer cells

**Figure 8:**
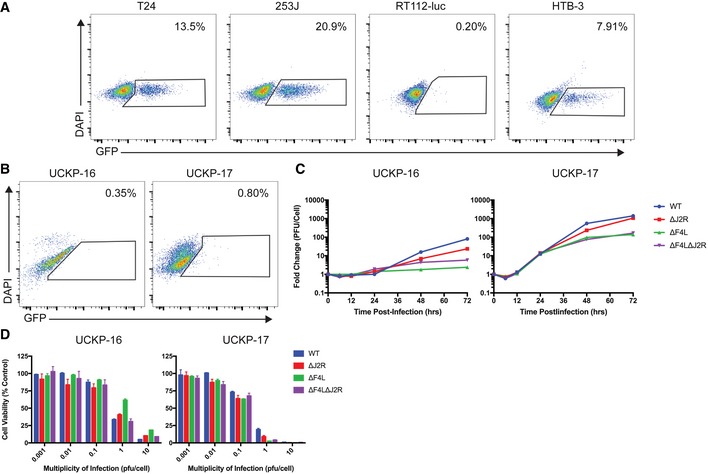
VACV replicates in a BCG‐resistant established cell line and in primary bladder tumor cultures BCG uptake in different cell lines. Cells were incubated with BCG‐GFP for 24 h, and then BCG uptake was measured by flow cytometry. The numbers show the percentage of GFP‐positive events out of total events. DAPI was used as an empty channel and the gates were set based on uninfected cells.BCG uptake by primary bladder tumor cultures. UCKP‐16 represents a low‐grade Ta tumor and UCKP‐17 represents a low‐grade T1 tumor. Cells were treated and analyzed as in (A).Growth of the indicated VACV strains in subconfluent primary human bladder tumor cultures *in vitro*. Cells were infected at a multiplicity of infection of 0.03 PFU/cell, harvested at the indicated times, and titered on BSC‐40 cells.VACV killing of UCKP‐16 and UCKP‐17 cells. Subconfluent cells were infected at the indicated multiplicities of infection (PFU/cell), cultured for 3 days, and assayed for viability with resazurin dye. Uninfected cells were used as control.Data Information: Data in (D) are shown as the mean ± SEM of single lysates titered in duplicate.

Accumulating evidence shows that attachment and internalization of BCG are required for its anti‐tumor activity (Zhao *et al*, 2000; Redelman‐Sidi *et al*, 2013), which creates a problem when tumors become resistant to infection. We were interested to see whether BCG‐resistant bladder cancer cells might still be sensitive to VACV infection. We exposed bladder cancer cells in culture to a Pasteur strain of BCG expressing green fluorescent protein, and measured infection by flow cytometry. These studies showed that T24, 253J, and HTB‐3 cells are BCG‐sensitive while the RT112‐luc cells are resistant to BCG uptake (Fig 8A). Nevertheless, all viruses replicated in both BCG‐sensitive and BCG‐resistant cell lines (Fig 1A). We also examined BCG uptake in primary cultures prepared from low‐grade Ta (UCKP‐16) and low‐grade T1 (UCKP‐17) bladder tumors. Although both primary cultures were resistant to BCG (Fig 8B), they were still killed by VACV (Fig 8D). The cytotoxicity was similar to that seen in established cell lines (Fig 1C) despite the lower levels of replication (Fig 8C). Interestingly, while ∆*F4L*∆*J2R* VACV replicated to slightly lower levels than WT and ∆*J2R* viruses (Fig 8C), it is just as effective at killing these two primary bladder cancer samples, and performed better at killing UCKP‐16 cells than the ∆*F4L* VACV. Overall, these results highlight the potential for using oncolytic VACV, particularly the ∆*F4L*∆*J2R* variant, to treat BCG‐refractory bladder cancer.

## Discussion

Bladder cancer has not received much attention as a target for clinical trials of oncolytic virotherapy [reviewed in (Potts *et al*, 2012; Delwar *et al*, 2016)]. Currently, the most advanced clinical trial is one using CG0070, a conditionally replicating adenovirus. CG0070 has completed a phase I trial (Burke *et al*, 2012) and is presently being evaluated in patients with advanced NMIBC who have failed BCG and refuse a cystectomy. However, the efficacy of this treatment is yet to be determined. It is interesting to note that in 2001, four patients with MIBC were treated with smallpox vaccine (Dryvax) intravesically before cystectomy. Three out of the four patients remained disease‐free after 4 years, which highlights the potential of VACV as a durable treatment for bladder cancer (Gomella *et al*, 2001). It is difficult to judge whether one could perform such a study today using the WT Dryvax virus, but these intriguing results suggest that a VACV modified to enhance tumor specificity and reduce virulence might offer a superior therapy for bladder cancer.

Most oncolytic VACVs reported to date bear mutations that inactivate *J2R. J2R* encodes the viral thymidine kinase, a critical enzyme in the salvage pathway for nucleotide biosynthesis (Russell *et al*, 2012). Deleting the *J2R* gene has been shown to reduce virulence while allowing VACV replication in dividing cells (Buller *et al*, 1985). We have shown here that all our bladder cancer cell lines supported replication of ∆*J2R* VACV to nearly the same level seen in cells infected with WT VACV. Surprisingly, we could not detect cellular TK1 in the normal N60 cell line even though it supports robust ∆*J2R* VACV growth. This could be explained by the fact that thymidine triphosphate can also be produced from deoxyuridine‐5′‐monophosphate by thymidylate synthase and thymidylate kinase (Romain *et al*, 2001) and VACV encodes the latter enzyme.

To produce a safer and more tumor‐selective oncolytic VACV, we investigated deletion of *F4L*, a homolog of the *RRM2* gene encoding the small subunit of ribonucleotide reductase. We used a panel of bladder cancer cell lines and primary tissues (Fig 2) to confirm reports that RRM2 is elevated in bladder cancer (Morikawa *et al*, 2010). Cancer cells often undergo metabolic reprogramming because of aberrant oncogenic signaling, adopting a state of anabolic metabolism to generate the needed macromolecules and dNTPs for division (Ward & Thompson, 2012). Additionally, cancer cells generally have an increased S‐phase fraction compared to normal cells (Aye *et al*, 2015). These characteristics of bladder tumor cells may partly explain the complementation of the viral RRM2 deletion, even under low serum conditions.

We observed robust replication of both ∆*F4L* VACV and ∆*F4L*∆*J2R* VACV in most bladder cancer cell lines and in BCG‐resistant RT112‐luc cells. Additionally, we have shown that ∆*F4L*∆*J2R* VACV can replicate in, and kill, BCG‐resistant primary bladder cancer cultures. Unlike a *J2R*‐deleted mutant, replication of the *F4L*‐deleted viruses was attenuated in non‐proliferating (partially serum‐deprived) normal cells.

For our initial *in vivo* experiments, we used both subcutaneous and orthotopic RT112‐luc tumors as models for NMIBC. This provides a replacement for HeLa‐contaminated KU7 or KU7‐luc cells (Jäger *et al*, 2013) and permitted bioluminescence monitoring of orthotopic tumor progression. RT112 cells have been used previously to model MIBC cancer by injecting cells into the bladder wall (Gust *et al*, 2013). However, MIBC is not treated by intravesical therapies. Our cell instillation technique produces a model for NMIBC, for which intravesical therapies are appropriate. These orthotopic RT112‐luc tumors responded dramatically to intravesical administration of the three mutant VACVs. In addition, intratumoral and intravenous injection of each of the three VACVs in the subcutaneous xenograft model produced tumor control in a manner that corresponded roughly to the degree of virus replication in the tumor. It is interesting to note that ∆*F4L*∆*J2R* VACV consistently produced better anti‐tumor activity than ∆*F4L* VACV in our animal models, and often *in vitro* as well. This seems counterintuitive, and the reason(s) why are unclear, but the mechanism is being investigated.

The VACV Western Reserve strain was originally adapted for growth in mice and exhibits virulence in this model. However, we saw no signs of toxicity in any of the immune‐compromised mice treated with ∆*F4L* or ∆*F4L*∆*J2R* VACV, while significant toxicity was observed in animals treated with ∆*J2R* VACV. In addition, ∆*J2R* VACV was recovered from multiple normal organs after intratumoral or systemic treatments. These data show that the *J2R* mutation does not suffice to prevent VACV replication in normal tissues of immune‐compromised mice. Although a number of oncolytic *J2R*‐deleted VACV strains have been used safely in many clinical trials, including one based on the VACV strain WR (Zeh *et al*, 2015), a further improvement might be obtained by incorporating *F4L* mutations. [In this regard, it is notable that Pexa‐Vec/JX‐594, a VACV lacking *J2R*, produced pox lesions in some patients (Cripe *et al*, 2015; Kung *et al*, 2015; Park *et al*, 2015)]. In another pre‐clinical study, Fend *et al* described an oncolytic WR strain bearing deletions in *J2R* and in *I4L*, which encodes the large subunit of ribonucleotide reductase (Fend *et al*, 2016). While this virus showed promise as a therapeutic agent, in our experience virus replication is regulated more stringently by *F4L* than by *I4L*, both *in vitro* and in mouse models (Gammon *et al*, 2010). This is likely due to RRM2 expression being cell‐cycle‐regulated whereas RRM1 is not (Gammon *et al*, 2010).

Our VACVs were also evaluated in an AY‐27 immune‐competent orthotopic rat model of bladder cancer. AY‐27 tumors resemble high‐grade human urothelial cell carcinoma (Xiao *et al*, 1999; Hendricksen *et al*, 2008) and an oncolytic reovirus has been previously tested in this model, where it proved more effective and less toxic than BCG (Hanel *et al*, 2004). In the current study, we saw that all rats treated with live VACV showed a reduction in tumor size, with complete tumor clearance in many animals, and a significant increase in survival relative to the controls. One caveat with the AY‐27 model is that up to 30% of the tumors invade the muscle by day 6 (Xiao *et al*, 1999; Hendricksen *et al*, 2008) and this characteristic may explain why some animals have a limited response to these therapies. While all three live VACV treatments produced statistically similar outcomes, it is interesting to note that regular monitoring of tumors by cystoscope showed that some of the rats treated with ∆*J2R* VACV had relapsed and this was not seen in rats treated with the *F4L*‐deleted VACVs. The ∆*J2R* VACV‐treated animals had significantly higher levels of neutralizing antibodies suggestive of a more robust anti‐viral, and thus more oncolysis‐limiting, response.

Outside of the previously cited studies, there has been little other pre‐clinical work with oncolytic viruses in immune‐competent bladder cancer models. Fodor *et al* studied a p53‐expressing *J2R*‐deleted VACV in an orthotopic MB49 immune‐competent mouse model and obtained three out of nine long‐term survivors (Fodor *et al*, 2005). OncoVEX^GALV/CD^, a herpes simplex virus type 1 engineered to express cytosine deaminase and a fusogenic glycoprotein, was tested in the AY‐27 rat bladder cancer model (Simpson *et al*, 2012) and caused a significant reduction in tumor volume. Unfortunately, in neither study was there any further investigation to establish whether treatment generated an anti‐tumor immune response.

As in the xenograft studies, the two *F4L*‐deleted VACVs proved highly tumor‐selective in the rat AY‐27 model. In contrast, ∆*J2R* VACV was detected in the lungs, kidneys, and ovaries in a subset of animals, although spread was not associated with overt toxicity. The ovaries consistently had the second highest levels of ∆*J2R* VACV after the tumor and we also saw what appeared to be ovarian cysts in multiple mice and rats. Other *J2R*‐deleted VACVs are reported to replicate in normal mouse tissues, including the ovaries where they can cause pathology and sterility (MacTavish *et al*, 2010; Zhao *et al*, 2011; Gentschev *et al*, 2012).

All rats that remained tumor‐free through day 125 (as determined by cystoscopy) exhibited anti‐tumor immunity as shown by tumor rejection upon challenge. *In vitro* assays performed 100 days after challenge confirmed the presence of long‐lasting tumor‐specific CD8^+^ T cells in all cured ∆*F4L*∆*J2R*‐treated rats. It is notable that these tumor‐specific cytotoxic T‐lymphocytes were only detected in animals that had also been exposed to live virus. Surprisingly, we know of no clear evidence that BCG can induce a protective anti‐tumor immune response (Ratliff *et al*, 1993; Gan *et al*, 1999). If BCG treatment is not also generating anti‐tumor immunity, it could explain the high recurrence rate in BCG‐treated patients.

As a final experiment, we showed that primary human bladder cancer tissues, in the form of either monolayers or as tumor fragments, could support the replication of both ∆*J2R* and ∆*F4L*∆*J2R* VACV *ex vivo* to a much greater degree than was seen in normal urothelium. This effect is similar to what has been reported for Pexa‐Vec (JX‐594) using rectal, endometrial, and colon cancer fragments along with adjacent normal tissues (Breitbach *et al*, 2011). The fact that ∆*F4L*∆*J2R* VACV also grew selectively in primary explanted human bladder cancer tissues provides some promise that these pre‐clinical results might also be seen in patients treated with oncolytic VACV.

In conclusion, these results suggest that NMIBC could be a highly suitable target for oncolytic treatment with a WR‐based ∆*F4L*∆*J2R* VACV and we are currently planning a phase I/II clinical trial with a similar VACV construct. From a practical perspective, intravesical bladder delivery offers a way of delivering high doses of virus directly to the tumor while also helping to limit systemic spread. ∆*F4L*∆*J2R* VACV showed an impressive safety profile, selectively infected a variety of susceptible cell types (including primary human bladder cancer tissues), and infection induced anti‐tumor immunity. Although patients with immune deficiencies and BCG‐refractory cancers would be ideal candidates for this therapy, in the longer‐term oncolytic ∆*F4L*∆*J2R* VACV might offer a more attractive replacement for BCG and potentially reduce the need for surgical management.

## Materials and Methods

### Cell lines

The human (253J, RT4‐luc, HTB‐3, HTB‐9, MGH‐U3, RT112‐luc, T24) and rat (AY‐27) bladder cancer cell lines were maintained in RPMI 1640 medium supplemented with 10% FBS, 2 mM l‐glutamine, 100 U/ml penicillin, 100 U/ml streptomycin, and 0.25 μg/ml Fungizone^®^ (Gibco). The HT‐1376, UM‐UC3‐luc, UM‐UC6, UM‐UC9, and UM‐UC14‐luc human bladder cancer cell lines were maintained in Dulbecco's modified Eagle's medium (DMEM)/F12 medium with the same supplements. MB49‐luc (murine urothelial cell carcinoma), N60 (early passage human skin fibroblast), NKC (normal human kidney epithelial), HeLa (CCL‐2), and RK3E (E1A immortalized rat kidney) cells were cultured in DMEM, also with the same supplements. BSC‐40 cells (CRL‐2761) were grown in minimal essential medium (MEM) supplemented as above, but using 5% FBS. Cells were cultured at 37°C in 5% CO_2_. RT4‐luc, RT112‐luc, and UM‐UC3‐luc cell lines were kindly provided by D. McConkey (MD Anderson), MB49 cells were kindly provided by J. Greiner (National Cancer Institute), and early passage N60 human skin fibroblast cells were kindly provided by T. Tredget (University of Alberta). All lines tested free of mycoplasma either by Hoechst 33342 staining (Thermo Fisher Scientific) and fluorescence imaging, or using a LookOut^®^ Mycoplasma PCR detection kit (Sigma‐Aldrich). The identities of the cell lines were confirmed using a 16‐marker AmpFLSTR^®^ Identifiler^®^ system and tests performed by the TCAG facility at the University of Toronto.

### Primary cell culture

Primary cancer and adjacent normal tissues were obtained from consenting patients undergoing surgery with the approval of the University of Alberta Health Research Ethics Board. Samples were received in saline and processed within 2 h of surgery. Submucosal and necrotic tissues were stripped from the tumor tissue and the remaining tumor was processed in 4 ml of “spleen dissociation medium” (STEMCELL Technologies) using a GentleMACS dissociator (Miltenyi Biotec). After rocking at 37°C for 30 min, the suspension was reprocessed, EDTA was added to 10 mM, and the cells were incubated at room temperature for 10 min. The cells were centrifuged for 5 min at 350 *g*, washed, and plated in EpiLife medium (Thermo Fisher Scientific) supplemented with 25 μg/ml bovine pituitary extract, 0.5 ng/ml epidermal growth factor, 3 mM glycine, 0.1 mM MEM non‐essential amino acids, 1% ITS (insulin, transferrin, selenium), 2% FBS, 2 mM l‐glutamine, 100 U/ml penicillin, 100 U/ml streptomycin, and 0.25 μg/ml Fungizone^®^ (Gibco).

### Biological agents

All recombinant viruses used in this study were derived from a clonal isolate of VACV strain WR, originally obtained from the American Type Culture Collection (ATCC). To permit *in vivo* imaging, we generated new versions of the ∆*F4L* and ∆*J2R* viruses described in Gammon *et al* (2010) that encode mCherry fluorescent protein under virus early/late promoter control. Detailed construction of the viruses is described in the Appendix Supplementary Methods.

GFP‐expressing BCG (BCG‐GFP) was a generous gift of Drs. Gil Redelman‐Sidi and Michael Glickman (MSKCC) and has been previously described (Redelman‐Sidi *et al*, 2013). Briefly, the BCG Pasteur strain was transformed with pYUB921 (episomal plasmid encoding GFP and conferring kanamycin resistance).

### *In vitro* infection experiments

Multistep growth curves were obtained by infecting the indicated cell lines with VACV at MOI of 0.03 PFU/cell. Infected cells were scraped into the culture medium at the indicated times, subjected to three rounds of freeze thaw and then diluted and plated in duplicate on BSC‐40 cells. Infected BSC‐40 cells were cultured in medium containing 1% carboxymethyl cellulose for 2 days and then fixed and stained with crystal violet. Plaque counts were determined from wells containing 30–250 plaques.

### Cytotoxicity assays

Cells were seeded in 48‐well plates at a density estimated to produce ~50% confluency at the time of infection. After an 8‐h incubation, cells were infected with VACV, cultured for 3 days and the media replaced with fresh cell culture media containing 44 μM resazurin (Sigma‐Aldrich). Plates were incubated 4–6 h at 37°C, and then fluorescence was read using a FLUOstar plate reader (BMG Labtech) with 560‐nm excitation/590‐nm emission filters.

### *In vivo* tumor models

All studies reported in this communication were conducted with the approval of the University of Alberta Health Sciences Animal Care and Use Committee in accordance with guidelines from the Canadian Council for Animal Care. Animals were housed with access to food and water *ad libitum* in ventilated mouse or rat cages (1–5 mice per cage, 1–2 rats per cage) in a biosafety level 2 containment suite at the University of Alberta Health Sciences Laboratory Animal Services Facility.

For all xenograft models, female Balb/c nude mice (Charles River Laboratories) were 8 weeks old and at least 16 g in weight at the time of tumor implantation. To establish orthotopic RT112‐luc tumors, mice were anesthetized with 2% isoflurane and a 27G angiocatheter (BD Biosciences), with the needle removed, was lubricated with sterile Lubrifax and inserted into the bladder *via* the urethra. The bladder was infused for 15 s with 50 μl of 0.1 M HCl, neutralized with 50 μl of 0.1 M KOH for 15 s, and then washed three times with phosphate‐buffered saline (PBS). Next, a 50 μl suspension of Hank's balanced salt solution (HBSS) containing 2 × 10^6^ RT112‐luc cells was instilled into the bladder using the catheter and left in‐dwell for 1 h while the mice remained under anesthesia. For virus treatments, the bladders of anesthetized mice were emptied by catheterization and then 50 μl of PBS, containing 1 × 10^6^ PFU of virus, was instilled into each mouse on days 10, 13, and 16 post‐implantations. The virus was left in‐dwell for 1 h while the mice remained under anesthesia.

To produce RT112‐luc or UM‐UC3‐luc flank tumors, Balb/c nude mice were anesthetized with isoflurane then injected subcutaneously with 0.1 ml of 2 × 10^6^ tumor cells in PBS containing 50% Matrigel (Corning).

Ten‐week‐old Fisher F344 immune‐competent female rats (Charles River Laboratories), weighing at least 150 g, were used for orthotopic AY‐27 tumor implantation as previously described (Xiao *et al*, 1999). Briefly, 0.3 ml 0.1 M HCl was instilled into the bladder of rats anesthetized with isoflurane, left in‐dwell for 15 s, and neutralized with 0.3 ml of 0.1 M KOH for 15 s, and then the bladder washed three times with PBS. The catheter was then used to deliver 0.3 ml of saline containing 3 × 10^6^ AY‐27 cells, the cells were left in‐dwell for 1 h, and the rats were returned to their cages. Five days later, tumor take was confirmed by cystoscopy (Asanuma *et al*, 2003). For virus treatments, the rats were anesthetized and catheterized, the bladders were emptied by suprapubic pressure, and then 3 × 10^8^ PFU of virus in 0.3 ml PBS was instilled into each bladder on days 6, 9, and 12 and left in‐dwell for 1 h.

Rats that were determined to be tumor‐free by cystoscopy at day 125 post‐tumor implantations were challenged with 3 × 10^6^ AY‐27 cells in the flank. Cells were resuspended in HBSS and mixed with equal volumes of Matrigel (Corning). A total of 200 μl was injected. Age‐matched Fisher F344 immune‐competent female rats were used as controls.

### Isolation of CD3^+^ cells

Spleens were harvested from euthanized rats and placed in HBSS on ice. Next, they were cut into small pieces, resuspended in 4 ml of spleen dissociation medium, and broken up using a GentleMACS dissociator, followed by rocking at 37°C for 30 min. The dissociation program was run again, EDTA was added to 10 mM, and the cells were incubated at room temperature for another 10 min. Cells were then filtered through a 70‐μm MACS SmartStrainer (Miltenyi Biotec), centrifuged for 5 min at 350 *g*, and washed with HBSS. Red blood cells were lysed, and then the remaining cells were recovered by centrifugation, then resuspended in PBS and counted. CD3^+^ cells were isolated from this preparation using a MagCellect™ Rat CD3^+^ T‐cell isolation kit following the manufacturer's protocol (R&D Systems).

### BMDC culture and lysate loading

The femurs were removed from euthanized naïve Fischer F344 female rats, cleaned of attached tissue, soaked in 70% isopropanol for 2 min, and rinsed in HBSS. Femur ends were removed and the marrow was flushed with HBSS. Red blood cells were lysed (eBioscience), and 3 × 10^6^ of the remaining bone marrow cells were plated on 100‐mm untreated plates in 8 ml RPMI 1640 medium supplemented with 10% FBS, 50 μM 2‐mercaptoethanol, 2 mM l‐glutamine, 100 U/ml penicillin, 100 U/ml streptomycin, 0.25 μg/ml Fungizone^®^ (Gibco), 500 U/ml rat GM‐CSF (Peprotech), and 20 ng/ml rat IL‐4 (Peprotech). The cells were cultured at 37°C and the medium, still containing GM‐CSF and IL‐4, was replaced on day 3. On day 5, the medium was replaced again, and 100 μg/ml of AY‐27 tumor lysate was added (see Appendix Supplementary Methods for preparation of tumor lysate). 12 h later, the cultures were matured by adding 20 U/ml TNF‐α (Peprotech) and 0.5 μg/ml CD40L (AdipoGen) (Labeur *et al*, 1999; Bachleitner‐Hofmann *et al*, 2002). Two days after adding the tumor cell lysates, the cultures were resuspended at 1 × 10^6^ cells/ml in fresh medium.

### T‐lymphocyte assays

Proliferation assays were performed in 96‐well U‐bottom plates (Greiner Bio‐One). BMDCs (pulsed with or without lysates) were co‐cultured with 10^5^ CD3^+^ cells at different ratios (1:1, 10:1, and 100:1 CD3:BMDC) in RPMI 1640 medium as described above. The CD3^+^ cells were previously labeled with CellTrace Violet per the manufacturer's directions (Thermo Fisher Scientific). After 6 days of co‐culture, flow cytometry was used to measure CD3^+^ T‐cell proliferation. Supernatants were collected from cells co‐cultured for 24 h, and assayed by ELISA for interferon‐γ (Legend Max, BioLegend™).

Cytotoxicity assays were performed using 10^5^ rat splenic CD3^+^ cells co‐cultured in 96‐well U‐bottom plates with 10^4^ BMDCs in RPMI 1640, supplemented as described above. On day 7, the CD3^+^ cells were collected and incubated for 18 h in flat‐bottom 96‐well plates, along with target cells, at effector‐to‐target ratios ranging from 20:1 to 0.625:1. The plates were assayed for lysis by LDH assay (Thermo Scientific Pierce). CD107a expression was measured essentially as described by Betts *et al* (2003). BMDCs were added to CD3^+^ cells, co‐cultured for 1 h in the presence of CD107a antibody, and incubated for another 5 h in the presence of monensin and brefeldin A (BioLegend). The cells were fixed and stained with anti‐CD4, anti‐CD8, and secondary to CD107a antibodies and analyzed by flow cytometry.

### Statistics

Data were analyzed using two‐tailed Student's *t‐*test when comparing the means of two groups. Multiple *t‐*test was used to determine significance of VACV growth after siRNA knockdown. Analysis of variance (ANOVA) was used when comparing multiple groups followed by Tukey's multiple comparison test. Microarray data were analyzed in the RStudio programming environment (v0.98.501). Significance analysis was performed by means of a one‐way ANOVA followed by Tukey's HSD. Data for animal survival curves were analyzed by log‐rank (Mantel–Cox) test. The numbers of animals included in each figure are indicated at the end of each legend. *P*‐values are indicated within each figure.

## Author contributions

KGP designed and performed experiments, analyzed the data, and prepared the manuscript. CRI designed the viruses used in these studies, performed the siRNA silencing experiment, and contributed to experimental design. NAF assisted in all animal experiments. RBM collected patient specimens and DBP and JDL performed *ex vivo* tumor imaging experiments and analyzed resulting data. KMV assisted in microarray data analysis. DHE, MMH, and RBM provided guidance in experimental design, data interpretation, and manuscript preparation.

## Conflict of interest

Dr. Evans has been awarded U.S. patents as a co‐inventor of related oncolytic virus technologies and is a co‐owner of Prophysis Inc., which retains a partial interest in the licensing rights for these technologies. The other authors declare that they have no conflict of interest.

**The paper explained**

**Problem**

Up to 80% of non‐muscle‐invasive bladder cancers (NMIBC) can recur within 5 years of initial treatment, with high‐grade NMIBC posing the greatest risk of recurrence. Treatment for these patients includes transurethral resection followed by intravesical therapy with the immunotherapeutic agent Bacillus Calmette–Guérin (BCG). BCG, however, carries the risk of systemic infection and can be particularly dangerous for immunocompromised patients. Additionally, up to 40% of patients fail BCG therapy and cystectomy remains the standard treatment in these cases. There is an urgent need for more bladder‐sparing therapies for patients failing conventional therapies.

**Results**

Here, we report the generation and testing of a novel oncolytic VACV that is lacking the viral *F4L* and *J2R* genes, homologs of cellular genes *RRM2* and *TK*, respectively, that promote nucleotide biosynthesis in infected cells. This oncolytic virus, ∆*F4L*∆*J2R* VACV, is highly attenuated in non‐cancerous cells and replicated selectively in both an orthotopic AY‐27 immunocompetent rat tumor model and an RT112‐luc xenografted human tumor model, causing significant tumor regression or complete tumor ablation with no toxicity. In contrast, a VACV with a more commonly employed deletion, ∆*J2R*, spread to normal organs and caused significant toxicity in immunocompromised mice. Furthermore, rats cured of AY‐27 tumors by VACV treatment developed a protective anti‐tumor immunity that was evidenced by tumor rejection upon challenge, as well as by *ex vivo* cytotoxic T‐lymphocyte assays. Finally, ∆*F4L*∆*J2R* VACV replicated in an established BCG‐resistant human bladder cancer cell line and in fresh cultures of primary human bladder tumors.

**Impact**

This study demonstrated the high degree of safety and anti‐tumor activity of a novel oncolytic virus in pre‐clinical bladder cancer models. Given the high rate of recurrence and the lack of treatment options for BCG‐resistant bladder cancer, our oncolytic VACV could provide a safe and urgently needed therapy for BCG failure.

## Supporting information



Appendix

Expanded View Figures PDF

Review Process File
