# Supplementary material for: Deletion of F4L (ribonucleotide reductase) in vaccinia virus produces a selective oncolytic virus and promotes anti‐tumor immunity with superior safety in bladder cancer models
Source: EMBO Mol Med. 2017 Mar 13;9(5):638–54. doi: 10.15252/emmm.201607296 (PMC5412795; doi:10.15252/emmm.201607296)
Supplement: Supplementary file 1 — Appendix [file EMMM-9-638-s001.pdf]

Deletion of *F4L* (ribonucleotide reductase) in vaccinia virus produces a selective oncolytic virus and promotes anti-tumor immunity with superior safety in bladder cancer models

Kyle G. Potts, Chad R. Irwin, Nicole A. Favis, Desmond B. Pink, Krista M. Vincent, John D. Lewis, Ronald B. Moore, Mary M. Hitt, David H. Evans

APPENDIX

Supplementary Methods
Supplementary Figures S1 – S7

APPENDIX SUPPLEMENTARY METHODS

*Viruses***.** Plasmid DNA encoding mCherry fluorescent protein under control of a synthetic early/late poxvirus promoter was subcloned from plasmid pE/L-mCherry-Topo2 into either pSC66 (to target the *J2R* locus) or R2-pZippyNeoGusA (to target *F4L*) (22). The resulting constructs were used to transfect Vero cells (ATCC CCL-81), 1 hr after infection with WT VACV at MOI=2 PFU/cell. The medium was replaced 2 hr later, the cells cultured for 48 hr, and the virus harvested by freeze-thaw. The mCherry-positive viruses were plaque purified using Vero cells. The virus referred to as ΔJ2R VACV encodes LacZ and mCherry genes disrupting *J2R*, and ΔF4L VACV encodes NeoGusA and mCherry disrupting *F4L* (See Figure EV1).

A third virus was produced by transfecting the R2-pZippyNeoGusA construct into cells infected with ΔJ2R VACV. This created ΔF4LΔJ2R VACV encoding NeoGusA disrupting *F4L,* and *LacZ* and mCherry genes disrupting *J2R*. The viruses were harvested, and recombinants detected by plating under an agar overlay containing 0.4mg/mL of 5-Bromo-4-chloro-3-indolyl-β-D-glucuronic acid (Clontech). PCR was used to confirm the purity of all the recombinant VACVs using primers 5'-TGACGTAAATGTGTGCGAAAGT-3' and 5'-TCAGCACCCATGA-ATGTCGAT-3' to amplify the F4L locus and primers 5'-TATTCAGTTGATAATCGGCCCC-ATGTTT-3' and 5'-GAGTCGATGTAACACTTTCTACACACCG-3' to amplify the J2R locus.

*BCG***.** BCG-GFP was grown in polystyrene roller bottles (Corning) at 37°C in Middlebrook 7H9 medium (Sigma-Aldrich) supplemented with 0.5% albumin, 0.2% dextrose, 0.085% saline, 0.5% glycerol (Thermo Fisher), 0.05% Tween 80 (Thermo Fisher), and 20 μg/ml kanamycin (Sigma-Aldrich). BCG-GFP was suspended in PBS with 25% glycerol and frozen at -80 °C. BCG-GFP titers were determined by spectrophotometric absorbance at 600 nm with 1 $OD_{600} = 5x10^8$ CFU-mL.

Antibodies. The primary antibodies used for western blots included: goat anti-RRM1 (Santa Cruz sc-1733), goat anti-RRM2 (Santa Cruz sc-10846), rabbit anti-p53R2 (Abcam ab8105), rabbit anti-TK1 (Abcam ab76495), rabbit anti-TK1 (Abcam ab59271), rabbit anti-β-actin (LICOR 926-42210), and rabbit anti-β-tubulin (CST #2416). The primary antibodies used for flow cytometry against rat included: mouse anti-CD4 FITC (eBiosciences 11-0040), mouse anti-CD8 APC (eBiosciences 17-0084), and mouse anti-CD107a (LAMP1) (Abcam ab2S630). The secondary antibodies used for western blots included: 680LT donkey anti-rabbit (LICOR 926-68023) and 800CW donkey anti-goat (LICOR 926-32214). The secondary antibody used for flow cytometry was donkey anti-mouse PE (Abcam ab7003).

Western blot analysis. Protein extracts were prepared from cells lysed on ice in buffer containing 150 mM NaCl, 50 mM Tris·HCl (pH 8.0), 0.5% sodium deoxycholate, 0.1% SDS, 1% NP-40, 0.1 mg/mL phenylmethylsulfonyl fluoride, and Halt protease inhibitor cocktail (Thermo Fisher). Protein extracts were prepared from human or animal tissues by adding 1 mL of the same buffer, but containing twice-concentrated protease inhibitors per 100 μg of tissue, and then homogenizing the suspension using a gentleMACS tissue dissociator (Miltenyi Biotec). Lysates were clarified by centrifugation and assayed for protein using a BCA protein assay kit (Thermo Fisher). For western blots, up to 30 μg of protein was fractionated by SDS-PAGE and transferred to Immobilon-FL PVDF membrane (EMD Milipore). The membranes were blocked with Odyssey blocking buffer (Li-COR Biosciences), diluted 1:1 with PBS for 1 hr at room temperature, and incubated overnight at 4°C with primary antibodies diluted in blocking buffer. Fluorescent-conjugated secondary antibodies were diluted in PBS containing 0.1% Tween and 0.01% SDS, and incubated with the membranes at room temperature for 1 hr. The washed membranes were

scanned using an Odyssey scanner (Li-COR Biosciences) and the images analyzed using ImageStudioLite software (Li-COR Biosciences).

*siRNA knockdown***.** 12 well plates were seeded with 100,000 HeLa (CCL-2) cells and 24 hr later were transfected with 20nM AllStars non-targeting siRNAs (Qiagen Cat#1027280) or RRM2 (hs RRM2 5 cat# S102653441) using Dharmafect 4 (GE Dharmacon) as per manufacturer's instructions.

Cell cycle analysis. Cells were harvested, pelleted by centrifugation for 5 min at 350 x g, washed once in PBS then re-centrifuged. Next, 70% ice-cold ethanol (2-3 mL) was added drop-wise to the cell pellets with continuous vortex-mixing. Cells were kept at 4°C for 30 min and then centrifuged at 1000 × g and 4°C for 5 min to remove the ethanol. The cell pellets were washed twice with PBS and resuspended in 0.5 mL of 0.50 µg/mL PI (Thermo Fisher) plus 10 µg/mL RNaseA (Thermo Fisher) and incubated at 37°C for 30 minutes. Cells were then analyzed by flow cytometry.

Preparation of tumor cells lysates. Cells were washed with PBS and then removed from the tissue culture plate using cell lifters (Thermo Fisher). Cells were the subject to five freeze (liquid nitrogen) and thaw (37°C water bath) cycles. To remove large particles, the lysate was centrifuged (2000 x g for 10 min at 4°C). Protein concentration was then determined using a BCA protein assay kit (Thermo Fisher).

Quantitation of VACV neutralizing antibodies. Sera were diluted in PBS and incubated with 500 PFU of VACV for 1 hr at 37°C. Next, the viruses were used to infect BSC-40 cells in 60 mm plates, then 48 hr later, cultures were stained with crystal violet and plaques counted.

Ex vivo infection of tumor explants. Virus-infected tumor tissues were imaged ~24 hr post-infection using a Zeiss Lumar stereomicroscope equipped with a Hamamatsu digital camera and

controlled with Volocity image acquisition software (PerkinElmer™). The same exposure and contrast settings were used for all tissues. Volocity image analysis software was used to quantify the mCherry signal and mock-infected tissue was used to establish a background.

Bioluminescence and fluorescence imaging. For bioluminescence imaging, mice bearing RT112-luc tumors were given an intraperitoneal injection of a solution containing 0.15 mL of 15 mg/mL D-Luciferin (Gold Biotechnology) in PBS. 12-15 min later, mice were imaged using an IVIS Spectrum imager (Caliper Life Sciences). White-light photographs and bioluminescence images were superimposed using Living Image software (Caliper Life Sciences, v 4.2). An average radiance was determined by manually selecting the tumor center with the Auto1 "region of interest" tool and using a threshold of 5%.

Fluorescence images were taken using the IVIS Spectrum imager (Caliper Life Sciences). All imaging utilized the built-in Image Wizard feature of Living Image software (Caliper Life Sciences, v 4.2). Images were acquired using the mCherry Spectral Unmixing spectrum (Excitation: 570 nM and Emission: 620, 640, 660, 680, 700, and 720 nm). A 500 nm excitation wavelength was also used for auto-fluorescence correction.

BCG infections. BCG infections were performed as described by Redelman-Sidi *et al*. (7). Bladder cancer cells were washed and held in serum and antibiotic-free medium for 1 hr prior to infection. A GFP-tagged strain of BCG (gift of Drs. Redelman-Sidi and Glickman) was added at a multiplicity of infection (MOI) of 5 CFU/cell, then the cells and bacteria were incubated at 37^oC for 24 hrs. The plates were washed three times with PBS, three times with serum and antibiotic-containing medium, and once with PBS. Next, the cells were detached with trypsin and resuspended in FACS buffer for analysis by flow cytometry.

Microarray datasets. Bladder cancer patient microarray expression data (log2 MAS 5.0 normalized Affymetrix U133A human GeneChip values) were retrieved from Sanchez-Carbayo M., *et al.* (26). Probes were selected for *RRM*1, *RRM2*, and *TK1* expression analysis: 201477_s_at, 209773_s_at, and 202338_at (respectively). For genes with more than one probe available (*RRM1*), the probe with the highest median expression value was chosen for analysis.

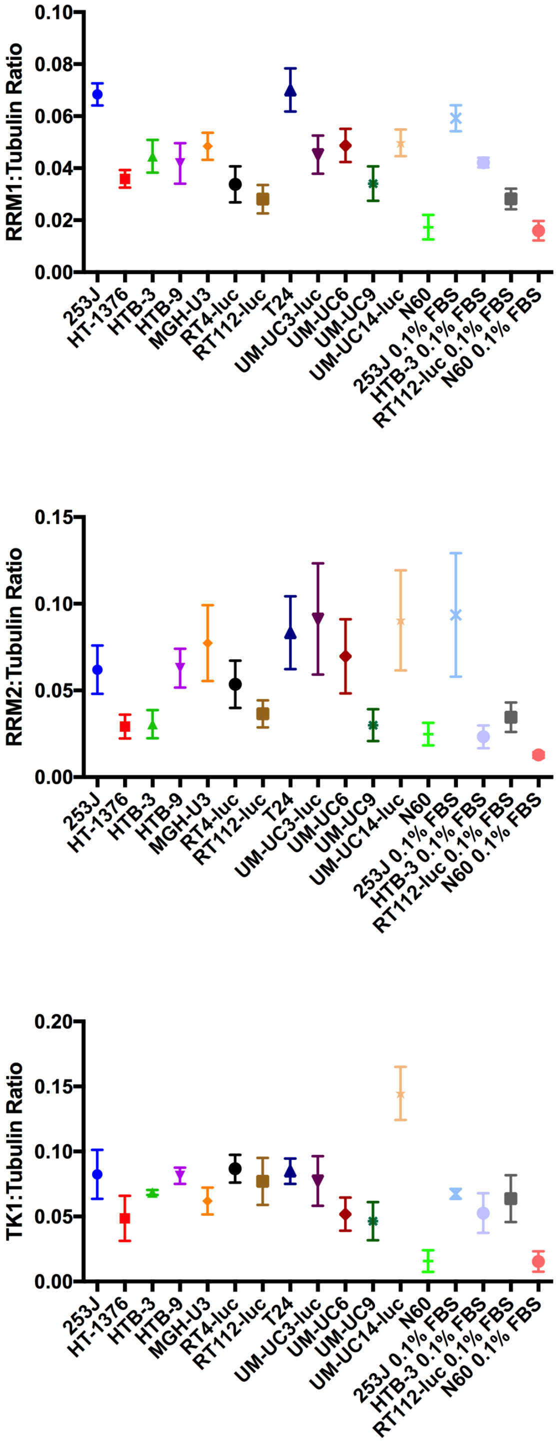

Appendix Figure S1: Quantification of levels of nucleotide metabolism proteins in bladder cancer cell lines. Quantification of RRM1, RRM2, p53R2, and TK1 expression relative to beta-tubulin (from Figure 2). The images were scanned using the LI-COR Odyssey scanner and quantified using Image Studio software (LI-COR Biosciences). The data are expressed as ratios relative to total beta-tubulin (mean ± SEM) of three independent experiments.

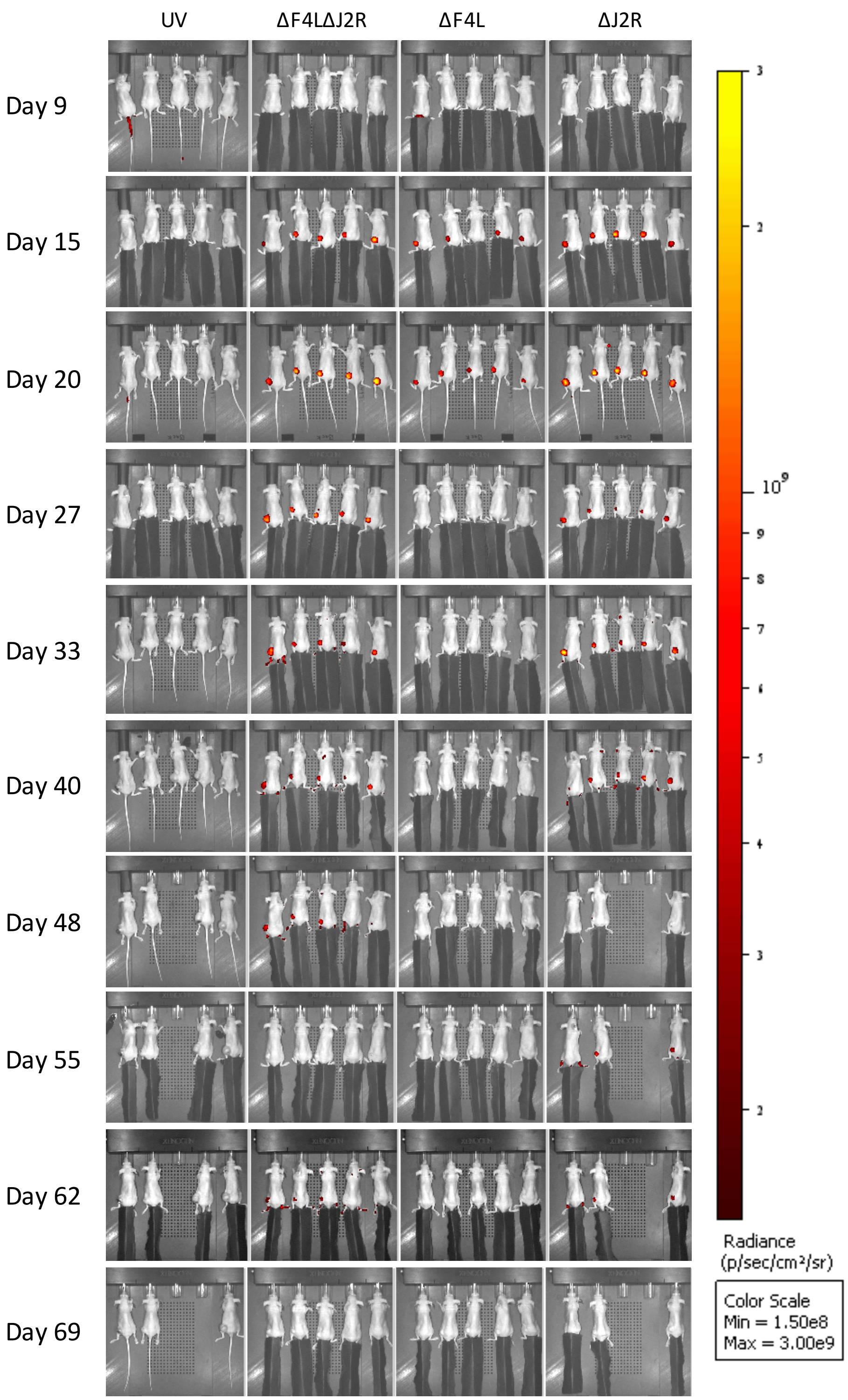

Appendix Figure S2: mCherry signal is detectable in RT112-luc subcutaneous xenografts after intratumoral injection of VACVs. Balb/c nude mice were injected with $2x10^6$ RT112-luc cells in the left flank on day 0. UV-inactivated, ΔJ2R, ΔF4L, and ΔF4LΔJ2R VACVs were injected intratumorally on days 10, 13, and 16 ($1x10^6$ PFU per injection). Mice were imaged for mCherry signal on the indicated days.

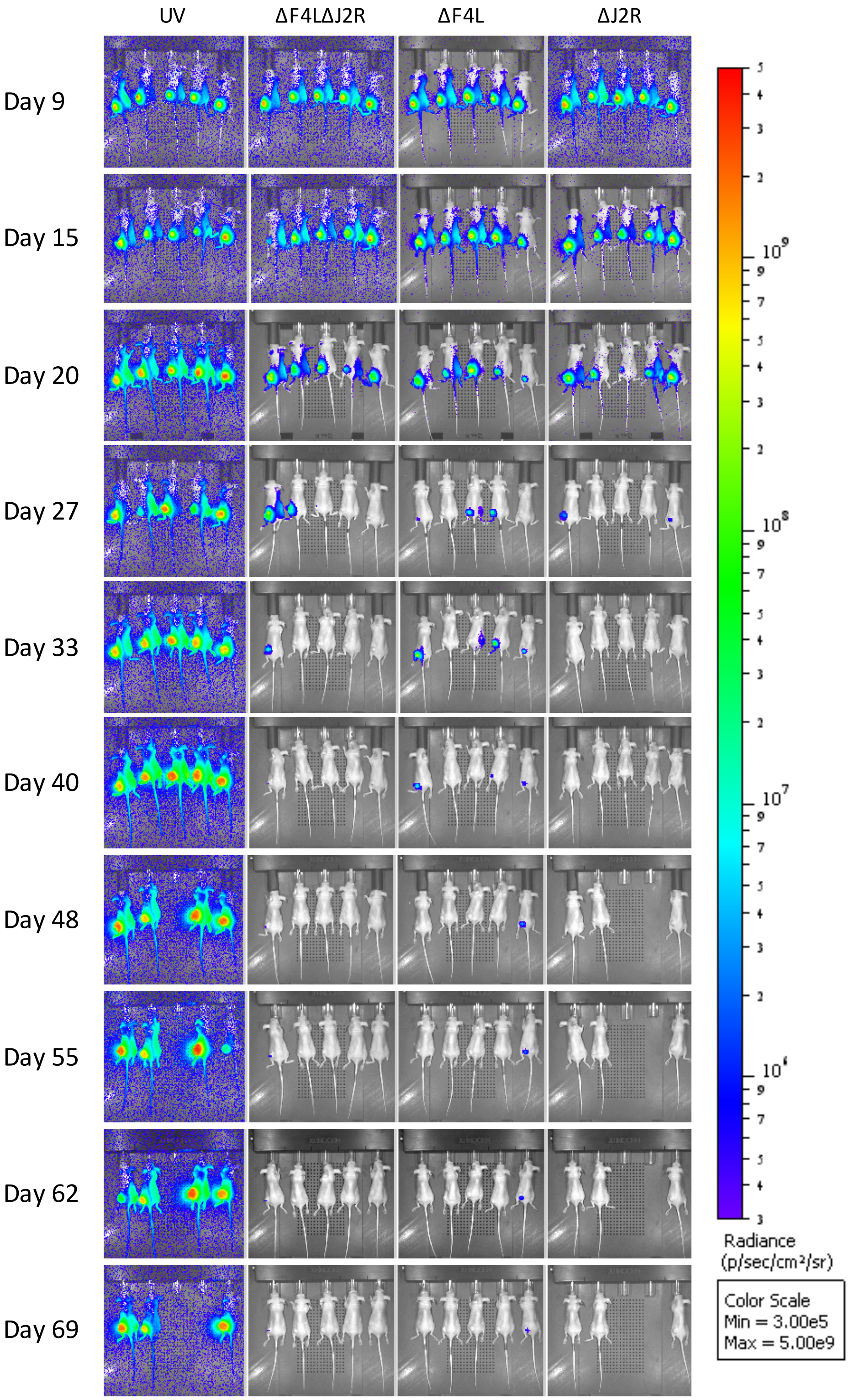

Appendix Figure S3: Intratumoral ΔF4LΔJ2R VACV effectively clears human RT112-luc subcutaneous xenograft tumors as indicated by luciferase signal. RT112-luc-tumor-bearing mice from the experiment shown in Figure S5 were imaged for luciferase expression following luciferin injection on the indicated days. All luciferase images are shown on the same scale.

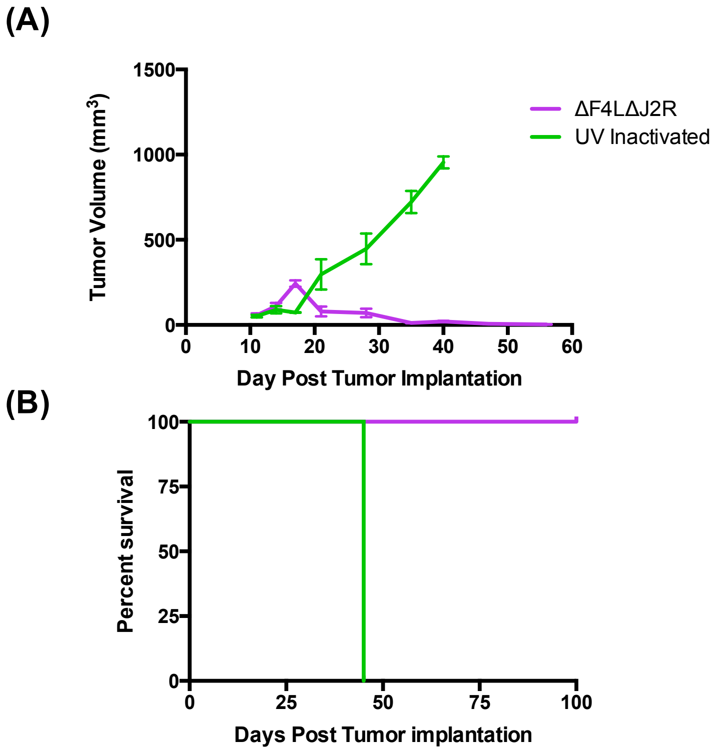

Appendix Figure S4: Intratumoral ΔF4LΔJ2R VACV safely and effectively clears human UM-UC3-luc subcutaneous xenograft tumors. Balb/c nude mice were injected with $2x10^6$ UM-UC3-luc cells in the left flank (day 0). UV-inactivatedor ΔF4LΔJ2R VACVs were injected intratumorally on days 10, 13, and 16 ($1x10^6$ PFU per injection). **(A)** Growth of VACV-treated UM-UC3-luc tumors. **(B)** Overall survival of immunocompromised mice bearing UM-UC3-luc flank tumors. Data represented in (A) and (B) obtained using n=3 mice per group and mean ± SEM is shown for (A).

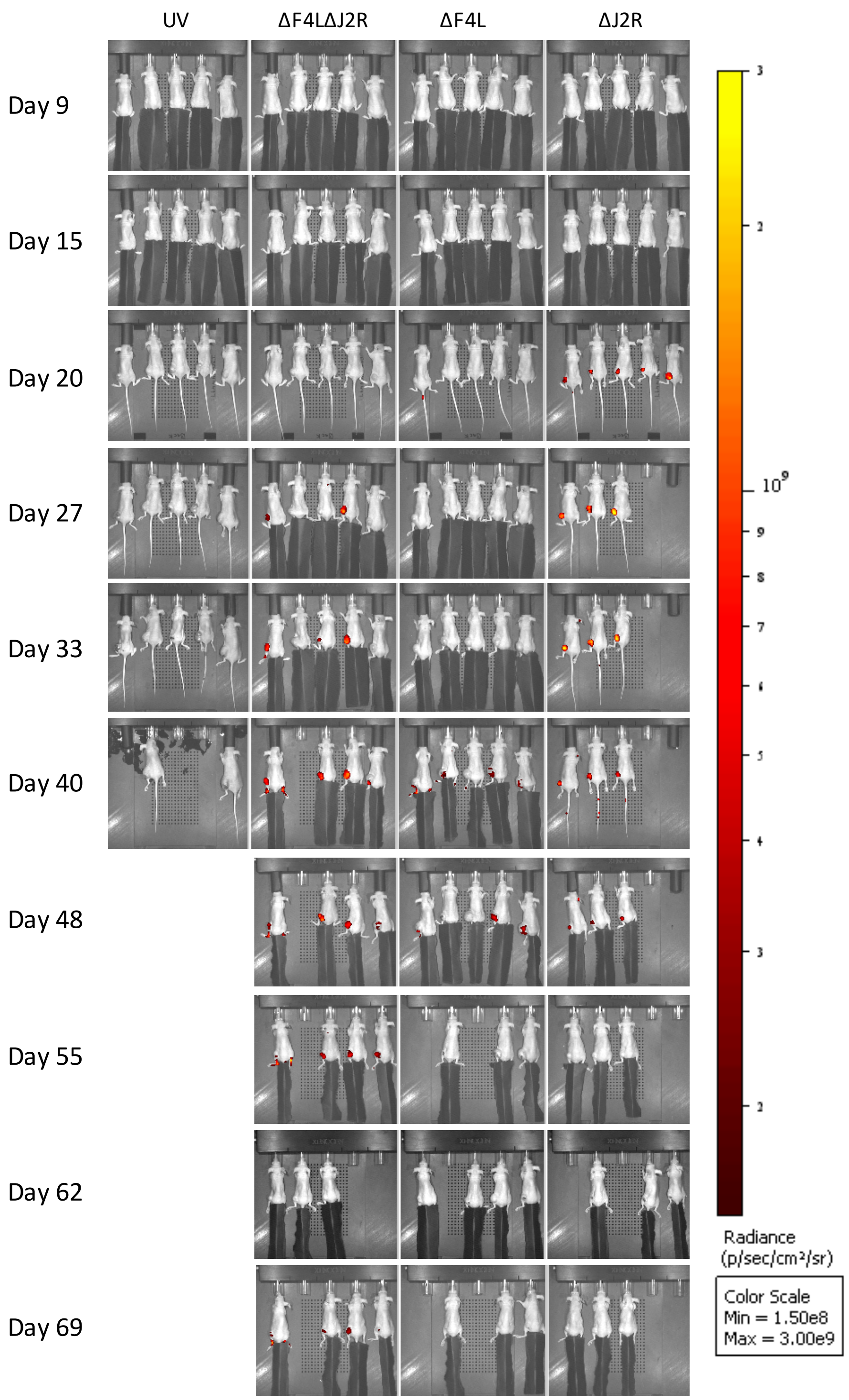

Appendix Figure S5: mCherry signal is detectable in RT112-luc xenografts after IV injection of VACVs. Mice were injected with $2x10^6$ RT112-luc cells in the left flank (day 0). $1x10^6$ PFU of UV-inactivated, ΔJ2R, ΔF4L, and ΔF4LΔJ2R VACVs were injected intravenously (via tail vein) on each of days 10, 13, and 16. Mice were imaged for mCherry signal on the indicated days.

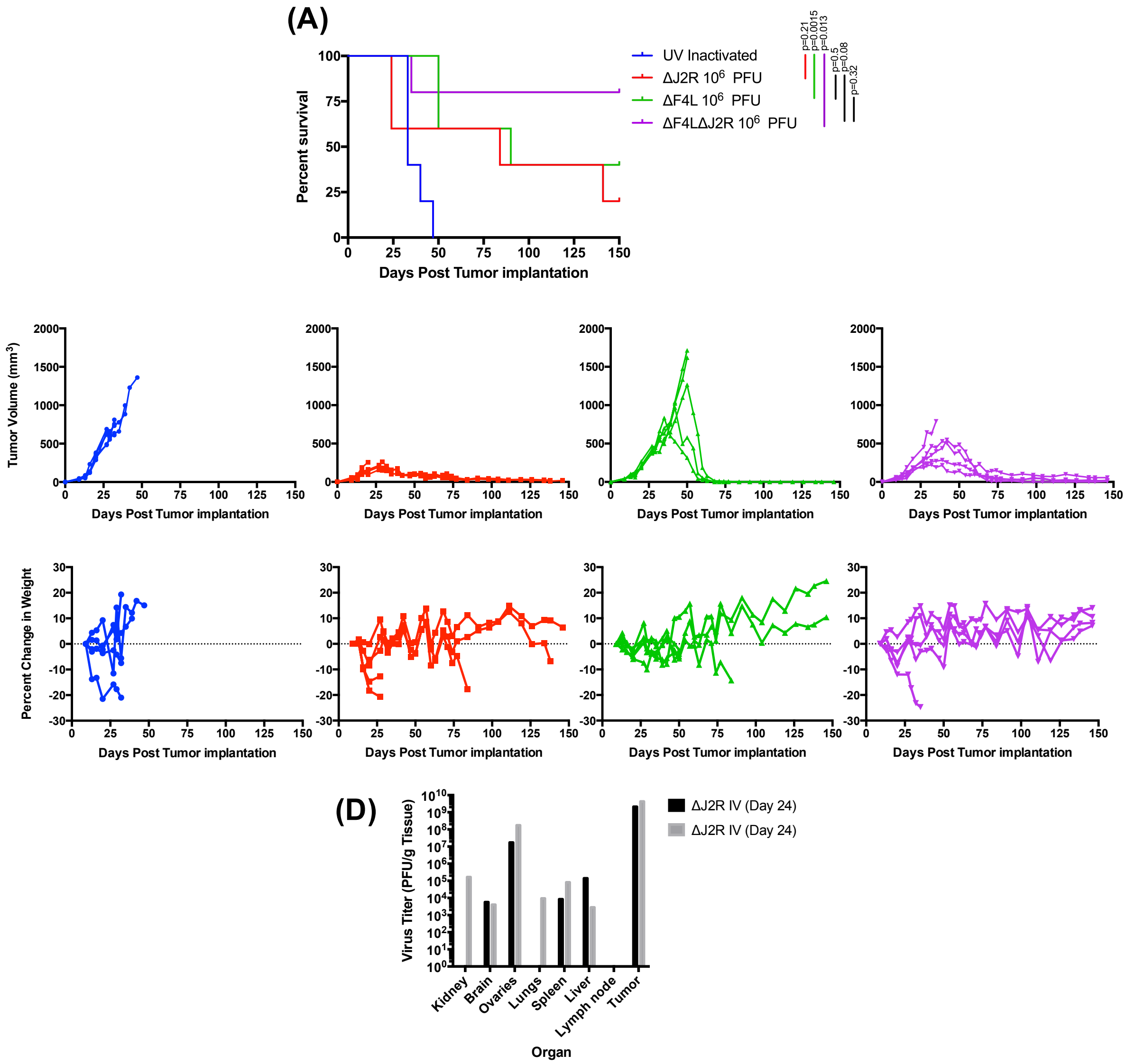

Appendix Figure S6: Intravenously injected ΔF4LΔJ2R VACV safely and effectively clears human RT112-luc xenografted tumors. Balb/c nude mice were injected with $2x10^6$ RT112-luc cells in the left flank (day 0). $1x10^6$ PFU of UV-inactivated, ΔJ2R, ΔF4L, and ΔF4LΔJ2R VACVs were injected by I.V. (via tail vein) on each of days 10, 13, and 16. **(A)** Overall survival of immunocompromised mice bearing RT112-luc flank tumors. **(B)** Growth of individual VACV-treated RT112-luc tumors. **(C)** Analysis of individual animals' body weight plotted as mean change in body weight. **(D)** VACV titers in tissues from euthanized animals (note: only mice that had detectable (2/4) virus as determined by plaque assay are shown). Organs were harvested and homogenized in HBSS using Miltenyi gentleMACS, then homogenates were titered on BSC40 cells. Data for (A) to (C) represent n=5. Animal survival was analyzed by log-rank (Mantel-Cox) test.

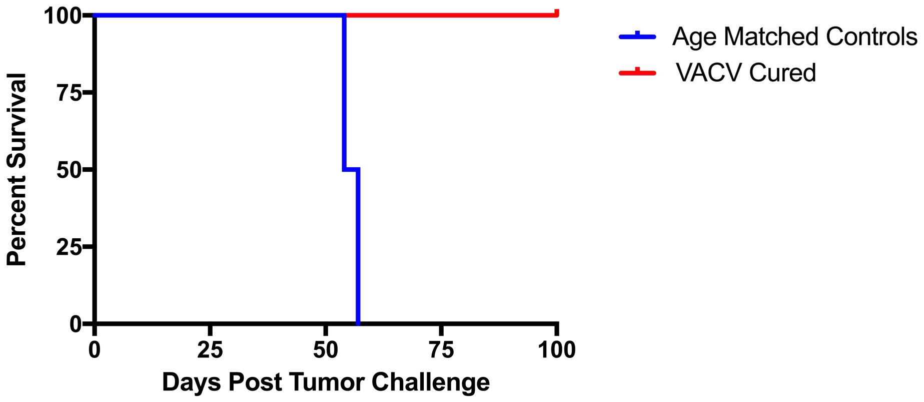

Appendix Figure S7: Protection from tumor challenge in bladder after virus-induced tumor clearance. AY-27 cells were implanted in the bladders of cured (n=11) and naïve (n=4) age-matched control rats. Cured animals consisted of 4 ΔJ2R, 2 ΔF4L, and 5 ΔF4LΔJ2R treated animals.
