# Supplementary material for: Deletion of F4L (ribonucleotide reductase) in vaccinia virus produces a selective oncolytic virus and promotes anti‐tumor immunity with superior safety in bladder cancer models
Source: EMBO Mol Med. 2017 Mar 13;9(5):638–54. doi: 10.15252/emmm.201607296 (PMC5412795; doi:10.15252/emmm.201607296)
Supplement: Supplementary file 2 — Expanded View Figures PDF [file EMMM-9-638-s002.pdf]

# Expanded View Figures

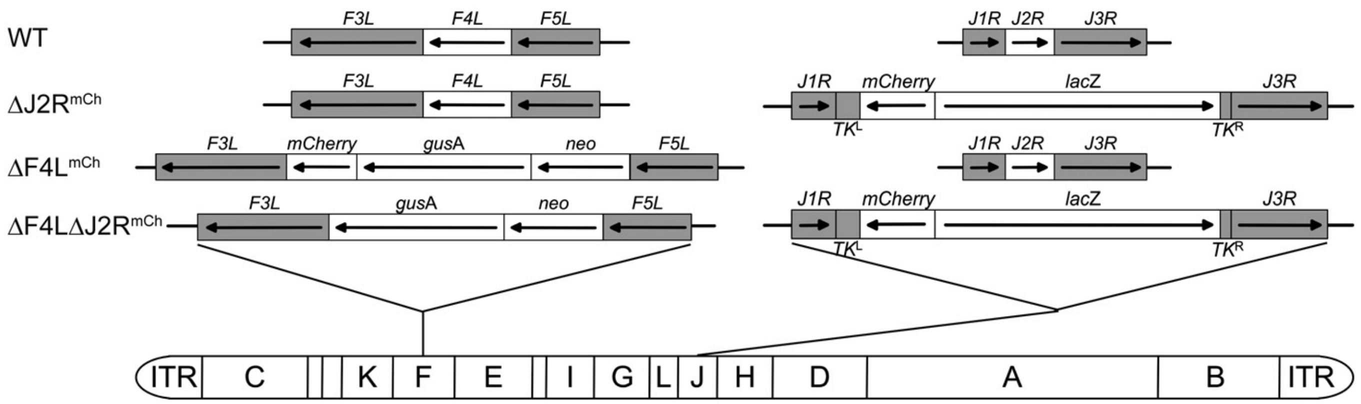


**Figure EV1. Genomic map of vaccinia virus constructs.**
Viruses were generated from the VACV Western Reserve strain. Viral thymidine kinase is encoded by the *J2R* gene. Subunit 2 of viral ribonucleotide reductase is encoded by the *F4L* gene. *neo*, neomycin gene; *gusA*, β-glucuronidase gene; *lacZ*, β-galactosidase gene; ITR, inverted terminal repeat; $TK^L$, viral thymidine kinase gene left homology; $TK^R$, viral thymidine kinase gene right homology; and WT, wild-type.

 © 2017 The Authors

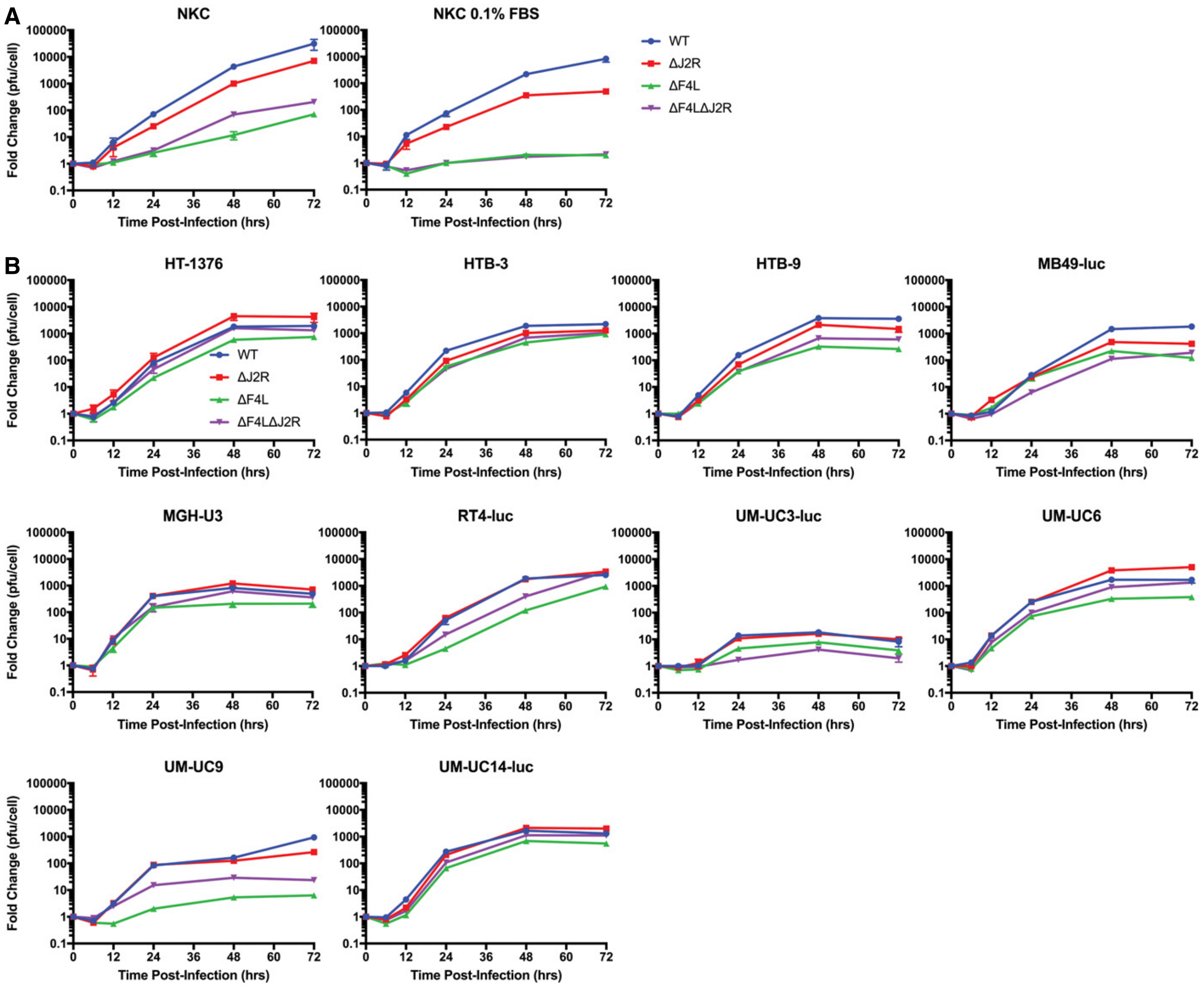


**Figure EV2. *ΔF4LΔJ2R* VACV retains much of the replication proficiency of wild-type VACV in bladder cancer cells.**
Growth curves for the indicated VACV mutants or WT VACV. Subconfluent cells were infected at a multiplicity of infection of 0.03 PFU/cell. Cultures were harvested at the indicated times and titered on BSC-40 cells.

A Normal human kidney epithelial cells grown under normal serum conditions (left) and 0.1% FBS (right).
B Panel of human bladder cancer cell lines (exception: MB49-luc, murine urothelial carcinoma) cultured *in vitro* with 10% FBS.

Data information: Mean ± SEM is shown and data represent at least two independent lysates titered in duplicate.

© 2017 The Authors

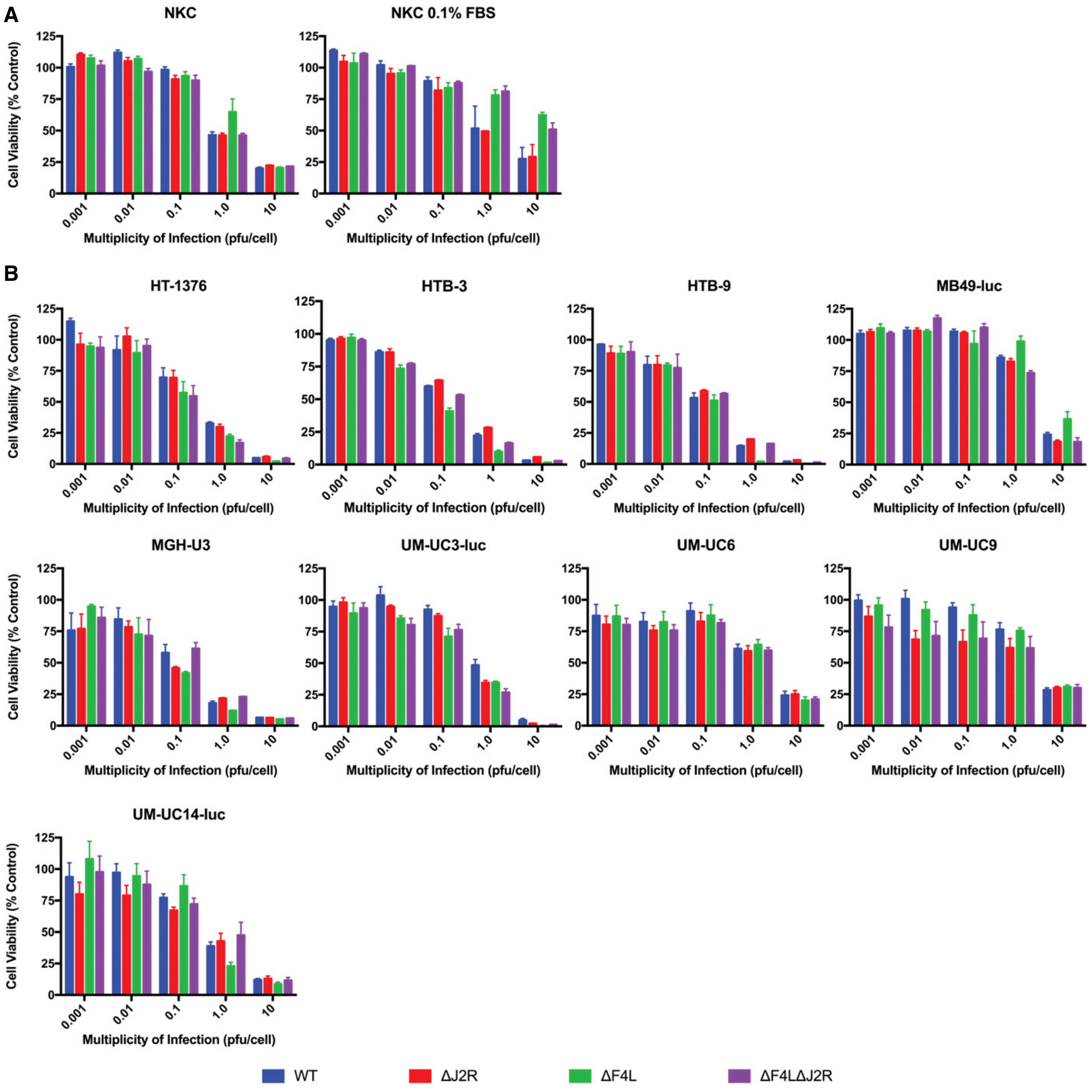


**Figure EV3. Δ*F4L*Δ*J2R* VACV retains much of the cytotoxicity of wild-type VACV in bladder cancer cells.**
Survival of cell lines infected *in vitro* with the indicated VACV strains. Subconfluent cells were infected at the indicated multiplicities of infection (in PFU/cell). Uninfected cells were used as control.

A Normal human kidney epithelial cells grown under normal serum conditions (left) and 0.1% FBS (right).
B Panel of human bladder cancer cell lines (exception: MB49-luc, murine urothelial carcinoma) cultured *in vitro* with 10% FBS. The cells were incubated with resazurin to assess viability 3 days post-infection relative to uninfected control cells. Uninfected cells were used as control.

Data information: Mean ± SEM is shown and $n \geq 3$.

 © 2017 The Authors

A

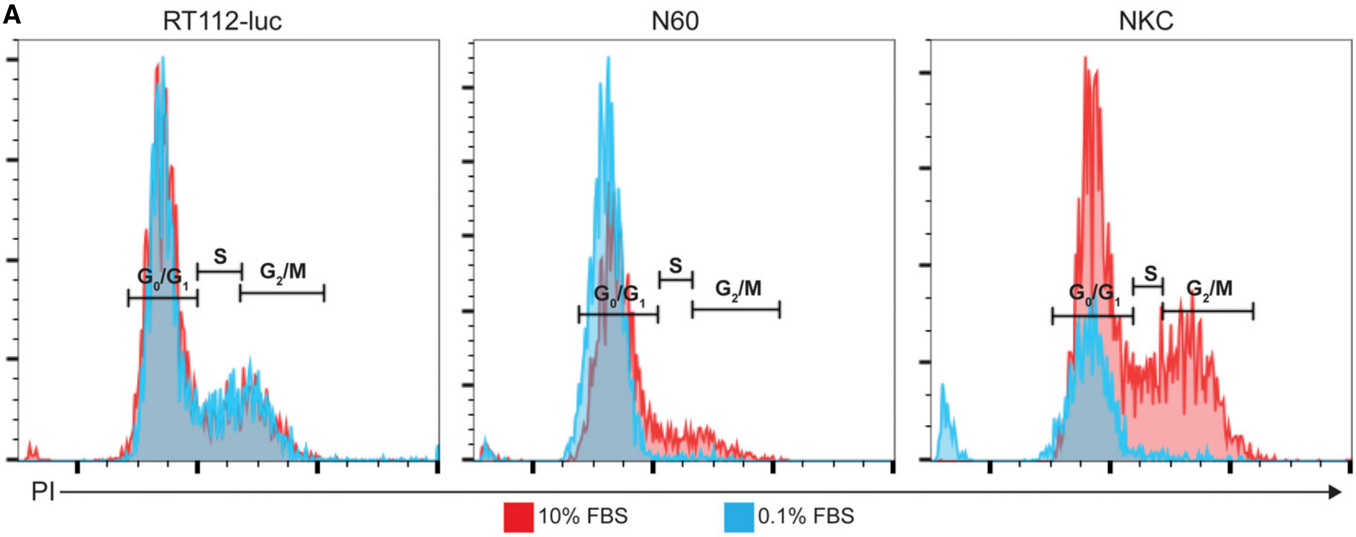


B

| Cell Line | $G_0/G_1$ | S | $G_2/M$ |
|---|---|---|---|
| RT112-luc 10% FBS | 67.1% | 15.3% | 16.7% |
| RT112-luc 0.1 % FBS | 70.2% | 12.1% | 16.0% |
| N60 10% FBS | 80.7% | 6.9% | 9.2% |
| N60 0.1% FBS | 93.4% | 1.0% | 1.5% |
| NKC 10% FBS | 58.8% | 10.6% | 28.9% |
| NKC 0.1% FBS | 79.4% | 2.8% | 3.6% |

**Figure EV4. Non-tumorigenic cells have reduced S-phase population when grown under lower serum conditions.**
Cell cycle analyses of indicated cell lines.

A Indicated cells lines were grown in media supplemented with either 10% FBS or 0.1% FBS for 48 h, and then cell cycle distribution was monitored by flow cytometry after PI staining. Red traces indicate cells grown in 10% FBS and blue traces indicate cells grown in 0.1% FBS.

B Analysis of cell cycle phase distribution.

© 2017 The Authors
